# Supplementary material for: The Systems Biology Research Tool: evolvable open-source software
Source: BMC Syst Biol. 2008 Jun 29;2:55. doi: 10.1186/1752-0509-2-55 (PMC2446383; doi:10.1186/1752-0509-2-55)
Supplement: Additional file 1 — SBRT Archive. An archive of the current version of the Systems Biology Research Tool. [file 1752-0509-2-55-S1.zip › sbrt-1.4.0/doc/developers_guide/api/index-files/index-7.html]

 
 
 
 
 
 
G-Index
 


 

 
function windowTitle()
{
    parent.document.title="G-Index";
}
 
 
 

 

 


 
   
  
 
 
 
   
 
   
          Overview     
        Package   
        Class   
        Use   
          Tree     
          Deprecated     
       Index    
          Help     
   
 
 
  
 
 
 

 
  
   PREV LETTER   
   NEXT LETTER    
  
    FRAMES     
   NO FRAMES     
  
    All Classes  ');
  }
  //-->
 
 
    All Classes  
 


  
 
 
  
 

 A   B   C   D   E   F   G   H   I   J   K   L   M   N   O   P   Q   R   S   T   U   V   W   X   Y   Z   
    
 G  
 
   GENE_FILE_NAME   - 
Static variable in class sbrt.shell.mng.fba. BiggSbmlReaderManager 
 Used to denote the name of the file to which gene-reaction relationships
 should be written.
   GENE_FILE_NAME   - 
Static variable in class sbrt.shell.mng.fba. BiggSbmlTranslaterManager 
 Used to denote the name of the file to which gene-reaction relationships
 should be written.
   GENE_FILE_NAME   - 
Static variable in class sbrt.shell.mng.fba. PalssonSbmlReaderManager 
 Used to denote the name of the file to which gene-reaction relationships
 should be written.
   GENE_FILE_NAME   - 
Static variable in class sbrt.shell.mng.fba. PalssonSbmlTranslaterManager 
 Used to denote the name of the file to which gene-reaction relationships
 should be written.
   get(int)   - 
Method in class sbrt.kernel.math.graph_theory. Path 
 Returns the node at the specified position in this path.
   get(int)   - 
Method in class sbrt.kernel.util. UnmodifiableList 
 See List.get(int).
   get(Object)   - 
Method in class sbrt.kernel.util. UnmodifiableMap 
 See Map.get(Object).
   get()   - 
Static method in class sbrt.shell.io. WorkingDirectory 
 Returns the current working directory.
   getActive()   - 
Method in class sbrt.kernel.fba.proc. RandomConstraintsGeneratorV1 
 Returns the constraint used to represent an active flux.
   getActive()   - 
Method in class sbrt.kernel.fba.proc. RandomEnvGeneratorV1 
 Returns the constraint used to represent an active source flux.
   getActive(ManagerInput<String, String>)   - 
Static method in class sbrt.shell.mng.fba. RandomConstraintsManager 
 Parses and returns the value for   RandomConstraintsManager.ACTIVE   in the 
 provided manager input.
   getActiveFluxes()   - 
Method in class sbrt.kernel.fba.proc. RandomConstraintsGeneratorV1 
 Returns the number of active fluxes in each set of randomly generated
 flux constraints.
   getActiveFluxes()   - 
Method in class sbrt.kernel.fba.proc. RandomEnvGeneratorV1 
 Returns the number of active source fluxes in each randomly generated
 environment.
   getAlwaysActive(Map<String, Interval>, double)   - 
Method in class sbrt.kernel.fba.analysis. FluxIntervalAnalyzer 
 Returns the always-active fluxes in the provided map.
   getAlwaysActive()   - 
Method in class sbrt.kernel.fba.proc. FluxPlasticityProcess.Results 
 Returns the set of fluxes that are always active.
   getApproxT()   - 
Method in class sbrt.kernel.math.stat.comp. MannWhitneyResults 
 Returns the t value approximated from the computed U statistic.
   getApproxZ(Map<? extends V, Double>, Map<? extends V, Double>)   - 
Method in class sbrt.kernel.math.stat.comp. KendallTau 
 Computes and returns a Z approximation for the provided values.
   getApproxZ(Collection<Double>, Collection<Double>)   - 
Method in class sbrt.kernel.math.stat.comp. KendallTau 
 Computes and returns a Z approximation for the provided values.
   getApproxZ(double[], double[])   - 
Method in class sbrt.kernel.math.stat.comp. KendallTau 
 Computes and returns a Z approximation for the provided values.
   getApproxZ()   - 
Method in class sbrt.kernel.math.stat.comp. TauResult 
 Returns the Z value approximated from the computed correlation statistic.
   getArray(Collection<? extends Number>)   - 
Static method in class sbrt.kernel.util. Util 
 Returns an array containing the values in the provided collection as doubles.
   getArray(Collection<Integer>)   - 
Static method in class sbrt.kernel.util. Util 
 Returns an array containing the values in the provided collection.
   getArray()   - 
Static method in class sbrt.shell.text. SpecialChars 
 Returns an array of the characters contained in this class.
   getArrays(Map<?, Double>, Map<?, Double>)   - 
Static method in class sbrt.kernel.util. Util 
 Returns a  2 x n  array containing the values from the provided
 maps.
   getBasic(String)   - 
Static method in class sbrt.shell.io. TextInputFile 
 Returns a basic text input file.
   getBasic(String, FileFormat)   - 
Static method in class sbrt.shell.io. TextInputFile 
 Returns a basic text input file.
   getBasic(String)   - 
Static method in class sbrt.shell.io. TextOutputFile 
 Returns a basic text output file.
   getBasic(String, FileFormat)   - 
Static method in class sbrt.shell.io. TextOutputFile 
 Returns a basic text output file with the provided
 file format.
   getBasic()   - 
Static method in class sbrt.shell.text. AmpersandSetFormat 
 Returns a  AmpersandSetFormat  that does not
 format or parse the elements supplied to it.
   getBasic()   - 
Static method in class sbrt.shell.text. BoolRelFormatV1 
 Returns an  BoolRelFormatV1  that does not format or parse the
 elements supplied to it.
   getBasic()   - 
Static method in class sbrt.shell.text. ColonMapEntryFormatV1 
 Returns a  ColonMapEntryFormatV1  that does not
 format or parse the keys or values supplied to it.
   getBasic()   - 
Static method in class sbrt.shell.text. ColonMapFormatV1 
 Returns a  ColonMapFormatV1  that does not
 format or parse the keys or values supplied to it.
   getBasic()   - 
Static method in class sbrt.shell.text. EqualsMapEntryFormatV1 
 Returns an  EqualsMapEntryFormatV1  that does not
 format or parse the keys or values supplied to it.
   getBasic()   - 
Static method in class sbrt.shell.text. EqualsMapFormatV1 
 Returns an  EqualsMapFormatV1  that does not
 format or parse the keys or values supplied to it.
   getBasic()   - 
Static method in class sbrt.shell.text.math. DirEdgeFormatV1 
 Returns a  DirEdgeFormatV1  that does not
 format or parse the nodes of the edges supplied to it.
   getBasic()   - 
Static method in class sbrt.shell.text.math. LinearCombFormatV1 
 Returns a  LinearCombFormatV1  that does not
 format or parse the variables supplied to it.
   getBasic()   - 
Static method in class sbrt.shell.text.math. LinearCombFormatV2 
 Returns a  LinearCombFormatV1  that does not
 format or parse the variables supplied to it.
   getBasic()   - 
Static method in class sbrt.shell.text. PipeListFormat 
 Returns a  PipeListFormat  that does not
 format or parse the elements supplied to it.
   getBasic()   - 
Static method in class sbrt.shell.text. PipeSetFormat 
 Returns a  PipeSetFormat  that does not
 format or parse the elements supplied to it.
   getBasic()   - 
Static method in class sbrt.shell.text. TabListFormat 
 Returns a  TabListFormat  that does not
 format or parse the elements supplied to it.
   getBasic()   - 
Static method in class sbrt.shell.text. TabSetFormat 
 Returns a  TabSetFormat  that does not
 format or parse the elements supplied to it.
   getBasic()   - 
Static method in class sbrt.shell.text. WhitespaceListFormat 
 Returns a  WhitespaceListFormat  that does not
 format or parse the elements supplied to it.
   getBasic()   - 
Static method in class sbrt.shell.text. WhitespaceSetFormat 
 Returns a  WhitespaceSetFormat  that does not
 format or parse the elements supplied to it.
   getBoundedMax(MathExpr<String>)   - 
Method in class sbrt.kernel.fba.comp. AbstractFluxIntervalComputer 
 Computes the maximum value of the provided objective function.
   getBoundedMax(String)   - 
Method in class sbrt.kernel.fba.comp. AbstractFluxIntervalComputer 
 Computes the maximum flux of the reaction with the provided name.
   getBoundedMaxWithReverseConstrained(String)   - 
Method in class sbrt.kernel.fba.comp. AbstractFluxIntervalComputer 
 Computes the maximum flux of the reaction with the provided name while
 constraining the fluxes of its reverse reactions to their minimum.
   getBoundedMin(MathExpr<String>)   - 
Method in class sbrt.kernel.fba.comp. AbstractFluxIntervalComputer 
 Computes the minimum value of the provided objective function.
   getBoundedMin(String)   - 
Method in class sbrt.kernel.fba.comp. AbstractFluxIntervalComputer 
 Computes the minimum flux of the reaction with the provided name.
   getBoundingPlanes()   - 
Method in interface sbrt.kernel.math.geom. ConvexPolytope 
 Returns the set of bounding hyperplanes that define this convex polytope.
   getBoundingPlanes()   - 
Method in class sbrt.kernel.math.geom. HashConvexPolytope 
 Returns the set of bounding hyperplanes that define this convex polytope.
   getBoundingPlanes()   - 
Method in class sbrt.kernel.math.geom. HashConvexPolytopeOld 
 Returns the set of bounding hyperplanes that define this convex polytope.
   getBounds()   - 
Method in exception sbrt.kernel.math. ConstraintViolationException 
 Returns the constraints that have been violated.
   getBounds()   - 
Method in class sbrt.kernel.math.geom. ParallelHyperplanes 
 Returns the bounding values used to define the inequality.
   getCanonicalName()   - 
Method in class sbrt.shell.io. AbstractMapInputFile 
 Returns the canonical name of this file.
   getCanonicalName()   - 
Method in class sbrt.shell.io. AbstractMapOutputFile 
 Returns the canonical name of this file.
   getCanonicalName(String)   - 
Static method in class sbrt.shell.io. AbstractSbrtFile 
 Returns the canonical name of the specified file.
   getCanonicalName()   - 
Method in class sbrt.shell.io.fba. ColumnOrFbaOptOutputFile 
 Returns the canonical name of this file.
   getCanonicalName()   - 
Method in class sbrt.shell.io.fba. FluxCapInputFile 
 Returns the canonical name of this file.
   getCanonicalName()   - 
Method in class sbrt.shell.io.fba. FluxIntervalInputFile 
 Returns the canonical name of this file.
   getCanonicalName()   - 
Method in class sbrt.shell.io.fba. RowOrFbaOptOutputFile 
 Returns the canonical name of this file.
   getCanonicalName()   - 
Method in class sbrt.shell.io.fba. SerFbaOptOutputFile 
 Returns the canonical name of this file.
   getCanonicalName()   - 
Method in interface sbrt.shell.io. SbrtFile 
 Returns the canonical name of this file.
   getCanonicalName()   - 
Method in class sbrt.shell.io. SerializedInputFile 
 Returns the canonical name of this file.
   getCanonicalName()   - 
Method in class sbrt.shell.io. SerializedOutputFile 
 Returns the canonical name of this file.
   getCanonicalName()   - 
Method in class sbrt.shell.io. TextInputFile 
 Returns the canonical name of this file.
   getCanonicalName()   - 
Method in class sbrt.shell.io. TextOutputFile 
 Returns the canonical name of this file.
   getCatalystRule(String)   - 
Method in interface sbrt.kernel.fba. CatalyzedFluxome 
 Returns the boolean logic rule for catalysts of the reaction with the 
 provided name.
   getCatalystRule(String)   - 
Method in interface sbrt.kernel.fba. IrrevRxnFluxome 
 Returns the boolean logic statement for the catalysts of the reaction
 with the provided name.
   getCatalystRule(String)   - 
Method in class sbrt.kernel.fba. IrrevRxnFluxomeV1 
 Returns the boolean logic statement for the catalysts of the reaction
 with the provided name.
   getCatalystRules()   - 
Method in interface sbrt.kernel.fba. CatalyzedFluxome 
 Returns the boolean logic rule for the catalysts of all reactions in
 this fluxome.
   getCatalystRules()   - 
Method in interface sbrt.kernel.fba. IrrevRxnFluxome 
 Returns all of the boolean logic statements regarding reaction catalysis.
   getCatalystRules()   - 
Method in class sbrt.kernel.fba. IrrevRxnFluxomeV1 
 Returns all of the boolean logic statements regarding reaction catalysis.
   getCatalysts()   - 
Method in interface sbrt.kernel.fba. CatalyzedFluxome 
 Returns the set of all catalysts contained in this fluxome.
   getCatalysts()   - 
Method in class sbrt.kernel.fba. IrrevRxnFluxomeV1 
 Returns the set of names of all catalysts contained in this fluxome.
   getCatalysts()   - 
Method in class sbrt.kernel.fba.proc. NetworkInfoProcess.Results 
 Returns the catalysts contained in the fluxome.
   getCatalysts(Fluxome, String)   - 
Static method in class sbrt.shell.mng.fba. FbaManager 
 Returns the reaction-catalyst associations contained in 
 the file with the provided name.
   getCatalysts(Fluxome, String, PrintWriter)   - 
Static method in class sbrt.shell.mng.fba. FbaManager 
 Returns the reaction-catalyst associations contained in the file with the
 provided name.
   getCatalysts(Node, String)   - 
Method in class sbrt.shell.text.fba. BiggRxnNodeParser 
 Returns the boolean logic rules for the catalysts of the provided
 reaction node.
   getCatalysts(Node, String)   - 
Method in class sbrt.shell.text.fba. PalssonRxnNodeParser 
 Returns the boolean logic rules for the catalysts of the provided
 reaction node.
   getCause()   - 
Method in exception sbrt.ext.mma. MathematicaException 
 Returns the  MathLinkException  responsible for this exception.
   getCentrality()   - 
Method in class sbrt.kernel.fba.comp. InitialPointComputer.Results 
 Returns the computed measure of centrality of the flux vector.
   getCentralityFile(ManagerInput<String, String>, PrintWriter)   - 
Static method in class sbrt.shell.mng.fba. SvInitialPointManager 
 Parses and returns the value for   SvInitialPointManager.AVG_P_FILE_NAME   in the provided
 manager input.
   getCentralityVector()   - 
Method in class sbrt.kernel.fba.comp. InitialPointComputer.Results 
 Returns the centrality vector.
   getChangedVariables()   - 
Method in class sbrt.kernel.math.geom.comp. CdHarComputer.Results 
 Returns the variables whose values are different than they were in
 the previous point.
   getChemSpecies()   - 
Method in class sbrt.kernel.fba.proc. NetworkInfoProcess.Results 
 Returns the metabolites contained in the fluxome.
   getChemSpeciesFormat()   - 
Method in class sbrt.shell.text.chem. IrrevRxnFormatV1 
 Returns the format used for chemical species.
   getChemSpeciesFormat()   - 
Method in class sbrt.shell.text.chem. IrrevRxnFormatV2 
 Returns the format used for chemical species.
   getChemSpeciesFormat()   - 
Method in class sbrt.shell.text.chem. RevRxnFormatV1 
 Returns the format used for chemical species.
   getChemSpeciesFormat()   - 
Method in class sbrt.shell.text.chem. RevRxnFormatV2 
 Returns the format used for chemical species.
   getChemSpeciesFormat()   - 
Method in class sbrt.shell.text.fba. IrrevRxnFormatV1 
 Returns the format used for chemical species.
   getCoeff()   - 
Method in class sbrt.ext.R.stat. RCorrelator.Result 
 Returns the computed correlation coefficient.
   getCoeff()   - 
Method in interface sbrt.kernel.math.stat.comp. CorrResult 
 Returns the computed correlation coefficient.
   getCoeff()   - 
Method in class sbrt.kernel.math.stat.comp. TauResult 
 Returns the computed correlation statistic.
   getCoeffFormat()   - 
Method in class sbrt.shell.text.math. LinCombTermFormatV1 
 Returns the format used for coefficients.
   getCoeffFormat()   - 
Method in class sbrt.shell.text.math. LinCombTermFormatV2 
 Returns the format used for coefficients.
   getCoeffFormat()   - 
Method in class sbrt.shell.text.math. LinearCombFormatV1 
 Returns the format used for coefficients.
   getCoeffFormat()   - 
Method in class sbrt.shell.text.math. LinearCombFormatV2 
 Returns the format used for coefficients.
   getCoefficient()   - 
Method in class sbrt.kernel.math. AbstractLinearComb.Term 
 Returns the coefficient of this term.
   getCoefficient(Object)   - 
Method in class sbrt.kernel.math. HashLinearComb 
 Returns the coefficient of the term containing the provided variable.
   getCoefficient(Object)   - 
Method in interface sbrt.kernel.math. LinearComb 
 Returns the coefficient of the term containing the provided variable.
   getCoefficient()   - 
Method in interface sbrt.kernel.math. Polynomial.Term 
 Returns the coefficient of this term.
   getColExprConstraint(MathExpr<?>)   - 
Method in class sbrt.kernel.fba.comp. FbaOptComputer 
 Returns the current constraint on the provided expression.
   getColExprConstraints()   - 
Method in class sbrt.kernel.fba.comp. FbaOptComputer 
 Returns all current constraints on mathematical expressions
 of column variables.
   getColExprs()   - 
Method in class sbrt.kernel.fba.comp. FbaOptComputer 
 Returns the set of all mathematical expressions of column variables.
   getColIndNzEl(Object)   - 
Method in class sbrt.kernel.math.algebra. HashMatrix 
 Returns the set of column indices whose elements are nonzero 
 in the row corresponding to the provided index.
   getColIndNzEl(Object)   - 
Method in class sbrt.kernel.math.algebra. RowOrTreeMatrix 
 Returns the set of column indices whose elements are nonzero 
 in the row corresponding to the provided index.
   getColIndNzEl(Object)   - 
Method in interface sbrt.kernel.math.algebra. SparseDoubleMatrix 
 Returns the set of column indices whose elements are nonzero 
 in the row corresponding to the provided index.
   getColIndNzEl(Object)   - 
Method in class sbrt.kernel.math.algebra. SparseDoubleMatrixWrap 
 Returns the set of column indices whose elements are nonzero 
 in the row corresponding to the provided index.
   getColIndNzEl(Object)   - 
Method in class sbrt.kernel.math.algebra. TreeMatrix 
 Returns the set of column indices whose elements are nonzero 
 in the row corresponding to the provided index.
   getColumn(Object)   - 
Method in interface sbrt.kernel.math.algebra. DoubleMatrix 
 Returns the column corresponding to the provided column index.
   getColumn(Object)   - 
Method in class sbrt.kernel.math.algebra. DoubleMatrixWrap 
 Returns the column corresponding to the provided column index.
   getColumn(Object)   - 
Method in class sbrt.kernel.math.algebra. HashMatrix 
 Returns the column corresponding to the provided column index.
   getColumn(Object)   - 
Method in interface sbrt.kernel.math.algebra. Matrix 
 Returns the column corresponding to the provided column index.
   getColumn(Object)   - 
Method in class sbrt.kernel.math.algebra. MatrixWrap 
 Returns the column corresponding to the provided column index.
   getColumn(Object)   - 
Method in class sbrt.kernel.math.algebra. RowOrTreeMatrix 
 Returns the column corresponding to the provided column index.
   getColumn(Object)   - 
Method in interface sbrt.kernel.math.algebra. SparseDoubleMatrix 
 Returns the column corresponding to the provided column index.
   getColumn(Object)   - 
Method in class sbrt.kernel.math.algebra. SparseDoubleMatrixWrap 
 Returns the column corresponding to the provided column index.
   getColumn(Object)   - 
Method in class sbrt.kernel.math.algebra. TreeMatrix 
 Returns the column corresponding to the provided column index.
   getColumnConstraint(Object)   - 
Method in class sbrt.kernel.fba.comp. FbaOptComputer 
 Returns the current constraint on the provided column variable.
   getColumnConstraints()   - 
Method in class sbrt.kernel.fba.comp. FbaOptComputer 
 Returns the current constraints for each column variable in
 the program matrix.
   getColumnHeaders()   - 
Method in class sbrt.shell.io.fba. ColumnOrFbaOptOutputFile 
 Returns the set of column headers that are written to
 this file.
   getColumnIndex()   - 
Method in class sbrt.kernel.math.algebra. IndexPair 
 Returns the column index.
   getColumnIndex()   - 
Method in class sbrt.kernel.math.algebra. MatrixElement 
 Returns the column index of this matrix element.
   getColumnIndexFormat()   - 
Method in class sbrt.shell.text.math. IndexPairFormatV1 
 Returns the format used for column indices.
   getColumnIndices()   - 
Method in class sbrt.kernel.math.algebra. HashMatrix 
 Returns the set of column indices contained in this
 matrix.
   getColumnIndices()   - 
Method in interface sbrt.kernel.math.algebra. Matrix 
 Returns the set of column indices contained in this
 matrix.
   getColumnIndices()   - 
Method in class sbrt.kernel.math.algebra. MatrixWrap 
 Returns the set of column indices contained in this
 matrix.
   getColumnIndices()   - 
Method in class sbrt.kernel.math.algebra. RowOrTreeMatrix 
 Returns the set of column indices contained in this
 matrix.
   getColumnIndices()   - 
Method in class sbrt.kernel.math.algebra. TreeMatrix 
 Returns the set of column indices contained in this
 matrix.
   getColumnMax(Object)   - 
Method in class sbrt.kernel.fba.comp. FbaOptComputer 
 Computes the maximum value of the provided column variable.
   getColumnMaxima(Collection<T>)   - 
Method in class sbrt.kernel.fba.comp. FbaOptComputer 
 Computes the maximum values of each provided column variable.
   getColumnMin(Object)   - 
Method in class sbrt.kernel.fba.comp. FbaOptComputer 
 Computes the minimum value of the provided column variable.
   getColumnMinima(Collection<T>)   - 
Method in class sbrt.kernel.fba.comp. FbaOptComputer 
 Computes the minimum values of each provided column variable.
   getColumnVariables()   - 
Method in class sbrt.kernel.fba.comp. FbaOptComputer 
 Returns the set of all column variables in the program matrix.
   getCommentChars()   - 
Method in class sbrt.shell.io. TextInputFile 
 Returns the commment characters used in this file.
   getCommonElements(Collection<? extends E>, Collection<? extends E>)   - 
Static method in class sbrt.kernel.util. Util 
 Returns a list of elements common to both provided collections.
   getComputer(ManagerInput<String, String>, PrintWriter)   - 
Static method in class sbrt.shell.mng.fba. CdHarFluxVectorManager 
 Creates and returns a coordinate-direction hit-and-run flux vector
 computer using the provided input.
   getComputer(ManagerInput<String, String>, PrintWriter, IrrevRxnFluxome, double)   - 
Static method in class sbrt.shell.mng.fba. CdHarFluxVectorManager 
 Creates and returns a coordinate-direction hit-and-run flux vector
 computer using the provided input.
   getComputer(ManagerInput<String, String>, PrintWriter)   - 
Static method in class sbrt.shell.mng.fba. ExtremeCurrentManager 
 Returns an extreme current computer created from the provided input.
   getComputer(ManagerInput<String, String>, PrintWriter)   - 
Static method in class sbrt.shell.mng.fba. FbaOptManager 
 Creates and returns an FBA optimization computer based on the provided
 input.
   getComputer(ManagerInput<String, String>, PrintWriter)   - 
Static method in class sbrt.shell.mng.fba. InitialPointManager 
 Creates and returns an FBA optimization computer based on the provided
 input.
   getComputer(ManagerInput<String, String>, PrintWriter)   - 
Static method in class sbrt.shell.mng.fba. MsFluxIntervalManager 
 Returns the flux interval computer specified by the provided input.
   getComputer(ManagerInput<String, String>, PrintWriter)   - 
Static method in class sbrt.shell.mng.fba. ReverseRxnFluxIntervalManager 
 Returns the flux interval computer specified by the provided input.
   getComputer(ManagerInput<String, String>, PrintWriter)   - 
Static method in class sbrt.shell.mng.fba. SimpleFluxIntervalManager 
 Returns the flux interval computer specified by the provided input.
   getConflicts()   - 
Method in exception sbrt.kernel.io. ConflictingInputException 
 Returns the list of conflicting inputs.
   getConstant()   - 
Method in class sbrt.kernel.math. AbstractPolynomial 
 Returns the constant of this polynomial.
   getConstant()   - 
Method in interface sbrt.kernel.math. MathExpr 
 Returns the numerical constant of this mathematical expression.
   getConstant()   - 
Method in interface sbrt.kernel.math. Polynomial 
 Returns the numerical constant of this polynomial.
   getConstraint(String)   - 
Method in class sbrt.kernel.fba. FluxConstraints 
 Returns the flux constraint for the provided reaction.
   getConstraint(MathExpr<String>)   - 
Method in class sbrt.kernel.fba. FluxConstraints 
 Returns the constraint of the provided mathematical expression of
 reaction names.
   getConstraints()   - 
Method in interface sbrt.kernel.fba. Fluxome 
 Returns the set of all constraints for fluxes and 
 mathematical expressions of fluxes in this fluxome.
   getConstraints()   - 
Method in class sbrt.kernel.fba. IrrevRxnFluxomeV1 
 Returns all constraints in this fluxome.
   getConstraints(ManagerInput<String, String>, PrintWriter, Fluxome)   - 
Static method in class sbrt.shell.mng.fba. FbaOptManager 
 Opens the file denoted by   FbaOptManager.CONSTRAINTS_FILE   in the provided
 manager input and returns the set of flux constraints it contains.
   getConstraints(String, Fluxome)   - 
Static method in class sbrt.shell.mng.fba. FbaOptManager 
 Returns the set of constraints contained in the file with the provided
 name.
   getConstraints(ManagerInput<String, String>, PrintWriter, Fluxome)   - 
Static method in class sbrt.shell.mng.fba. RandomFluxVectorManager 
 Opens the file denoted by   FbaOptManager.CONSTRAINTS_FILE   in the 
 provided manager input and returns the set of flux constraints 
 it contains.
   getConstraints(String, Fluxome)   - 
Static method in class sbrt.shell.mng.fba. RandomFluxVectorManager 
 Returns the set of constraints contained in the file with the provided
 name.
   getConstraintTolerance()   - 
Method in class sbrt.kernel.fba.comp. AbstractFluxIntervalComputer 
 Returns the constraint tolerance used by this flux interval computer.
   getConstraintTolerance()   - 
Method in class sbrt.kernel.fba.comp. CdHarFluxVectorComputer 
 Returns the constraint tolerance.
   getConstraintTolerance()   - 
Method in class sbrt.kernel.fba.comp. FastMsFluxRangeComputer 
 Returns the constraint tolerance used by this flux interval computer.
   getConstraintTolerance()   - 
Method in interface sbrt.kernel.fba.comp. FluxIntervalComputer 
 Returns the amount by which constraints can be violated.
   getConstraintTolerance()   - 
Method in class sbrt.kernel.math.geom.comp. CdHarComputer 
 Returns the constraint tolerance.
   getConstraintTolerance(ManagerInput<String, String>, PrintWriter)   - 
Static method in class sbrt.shell.mng.fba. FbaManager 
 Parses and returns the value for   FbaManager.CONSTRAINT_TOLERANCE   in the 
 provided manager input.
   getConstraintTolerance(ManagerInput<String, String>)   - 
Static method in class sbrt.shell.mng.fba. FbaManager 
 Parses and returns the value for   FbaManager.CONSTRAINT_TOLERANCE   in the 
 provided manager input.
   getConstraintTolerance(ManagerInput<String, String>, PrintWriter)   - 
Static method in class sbrt.shell.mng.geom. CdHarManager 
 Parses and returns the value for   CdHarManager.CONSTRAINT_TOLERANCE   in the
 provided manager input.
   getCorrName()   - 
Method in class sbrt.ext.R.stat. RCorrelator 
 Returns the name of the correlation statistic computed by this  R 
 correlator.
   getCorrName()   - 
Method in class sbrt.ext.R.stat. RKendall 
 Returns  kendall .
   getCorrName()   - 
Method in class sbrt.ext.R.stat. RPearson 
 Returns  pearson .
   getCorrName()   - 
Method in class sbrt.ext.R.stat. RSpearman 
 Returns  spearman .
   getCount()   - 
Method in class sbrt.kernel.util. AbstractProgressMonitor 
 Returns the number of times of  increment()  has been called.
   getCVectorFile(ManagerInput<String, String>, PrintWriter, Fluxome)   - 
Static method in class sbrt.shell.mng.fba. InitialPointManager 
 Returns an empty output file that is suitable for storing a single C
 vector with the name denoted by   InitialPointManager.P_VECTOR_FILE_NAME   in the
 provided input.
   getCvInputFile(ManagerInput<String, String>, PrintWriter, Fluxome)   - 
Static method in class sbrt.shell.mng.fba. IntervalVariationManager 
 Returns the constraint variation input with the name denoted by
   IntervalVariationManager.CV_FILE   in the provided manager input.
   getCvInputFile(String, Fluxome)   - 
Static method in class sbrt.shell.mng.fba. IntervalVariationManager 
 Returns the constraint variation input file with the provided name.
   getCycles(IrrevRxnFluxome)   - 
Method in interface sbrt.kernel.fba.comp. CycleComputer 
 Returns the set of all steady state cycles contained in the provided
 fluxome.
   getCycles(IrrevRxnFluxome)   - 
Method in class sbrt.kernel.fba.comp. SlpCycleComputer 
 Returns the set of all steady state cycles (Type III extreme pathways)
 contained in the provided fluxome.
   getCycles(ManagerInput<String, String>, PrintWriter, Fluxome)   - 
Static method in class sbrt.shell.mng.fba. FluxCapManager 
 Opens the file denoted by   FluxCapManager.CYCLE_FILE   in the 
 provided manager input and returns the list of cycles it contains.
   getDataHeaders(ManagerInput<String, String>, Fluxome)   - 
Static method in class sbrt.shell.mng.fba. FbaOptManager 
 Parses and returns the value for   AbstractProcessManager.DATA_HEADERS   in the provided
 manager input.
   getDataHeaders(String, Fluxome)   - 
Static method in class sbrt.shell.mng.fba. FbaOptManager 
 Parses the provided data header string and returns the corresponding set.
   getDefault()   - 
Static method in class sbrt.kernel.io. WarningHandlerFactory 
 Returns the default warning handler.
   getDeletionComputer(ManagerInput<String, String>, PrintWriter)   - 
Static method in class sbrt.shell.mng.fba. RxnDeletionManager 
 Returns a reaction deletion computer created from the provided input.
   getDeletionFile(String, CatalyzedFluxome)   - 
Static method in class sbrt.shell.mng.fba. CatalystDeletionManager 
 Returns the catalyst input file with the provided name.
   getDeletiontFile(ManagerInput<String, String>, PrintWriter, CatalyzedFluxome)   - 
Static method in class sbrt.shell.mng.fba. CatalystDeletionManager 
 Parses and returns the value for   CatalystDeletionManager.CATALYST_DELETION_FILE   
 in the provided manager input.
   getDelimiter()   - 
Method in class sbrt.shell.text. AbstractLinkedHashSetFormat 
 Returns the delimiter used to separate elements
 when producing a formatted string.
   getDelimiter()   - 
Method in class sbrt.shell.text. AbstractListFormat 
 Returns the delimiter used to separate elements when producing a
 formatted string.
   getDelimiter()   - 
Method in class sbrt.shell.text. AbstractMapEntryFormat 
 Returns the delimiter used to separate the key from the
 value in a key-value pair.
   getDiffs(Map<? extends V, Interval>, Map<? extends V, Interval>, double)   - 
Method in class sbrt.kernel.math. IntervalComparer 
  
   getDiffs(Map<? extends V, Double>, Map<? extends V, Double>, double)   - 
Method in class sbrt.kernel.math. VectorComparer 
  
   getDimension()   - 
Method in exception sbrt.kernel.math.geom. DimensionException 
 Returns the invalid dimension.
   getDirection()   - 
Method in exception sbrt.kernel.math. InfeasibleSolutionException 
 Returns the variable, or direction in hyperspace, in which infeasibility
 was first detected.
   getDist(Number[], Number[])   - 
Method in class sbrt.kernel.math.geom.comp. EuclideanDist 
 Computes and returns the Euclidean distance between the provided arrays.
   getDist(Collection<? extends Number>, Collection<? extends Number>)   - 
Method in class sbrt.kernel.math.geom.comp. EuclideanDist 
 Computes and returns the Euclidean distance between the provided collections.
   getDist(Map<?, ? extends Number>, Map<?, ? extends Number>)   - 
Method in class sbrt.kernel.math.geom.comp. EuclideanDist 
 Computes and returns the Euclidean distance between the provided maps.
   getDist(String, String)   - 
Method in class sbrt.kernel.math.geom.comp. Hamming 
 Computes and returns the Hamming distance between the provided strings.
   getDist(Object[], Object[])   - 
Method in class sbrt.kernel.math.geom.comp. Hamming 
 Computes and returns the Hamming distance between the provided arrays.
   getDist(Collection<?>, Collection<?>)   - 
Method in class sbrt.kernel.math.geom.comp. Hamming 
 Computes and returns the Hamming distance between the provided collections.
   getDist(Map<?, ?>, Map<?, ?>)   - 
Method in class sbrt.kernel.math.geom.comp. Hamming 
 Computes and returns the Hamming distance between the provided maps.
   getDist(Number[], Number[])   - 
Method in class sbrt.kernel.math.geom.comp. Taxicab 
 Computes and returns the Taxicab distance between the provided arrays.
   getDist(Collection<? extends Number>, Collection<? extends Number>)   - 
Method in class sbrt.kernel.math.geom.comp. Taxicab 
 Computes and returns the Taxicab distance between the provided collections.
   getDist(Map<?, ? extends Number>, Map<?, ? extends Number>)   - 
Method in class sbrt.kernel.math.geom.comp. Taxicab 
 Computes and returns the Taxicab distance between the provided maps.
   getDOM(String)   - 
Static method in class sbrt.shell.io. XmlFileFactory 
 Returns a DOM object for the file with the specified name.
   getDoubleFormat()   - 
Method in class sbrt.shell.text.fba. FbaOptFileLineFormatV1 
 Returns the format used for doubles.
   getDoubleFormat()   - 
Method in class sbrt.shell.text.math. BoundedIntervalFormatV1 
 Returns the double format used for bounds.
   getDoubleFormat()   - 
Method in class sbrt.shell.text.math. IntervalFormatV1 
 Returns the double format used for bounds.
   getDoubleFormat()   - 
Method in class sbrt.shell.text. PercentFormatVersion1 
 Returns the  DoubleFormat  used to 
 parse and format the numerical percentage values.
   getDoubleTester()   - 
Method in class sbrt.kernel.math. IntervalEquiv 
 Returns the double equivalence tester used by this interval equivalence tester.
   getDuplicates(Collection<E>)   - 
Static method in class sbrt.kernel.util. Util 
 Returns a list of duplicate elements contained in the provided
 collection.
   getDuration()   - 
Method in class sbrt.kernel.util. AbstractProgressMonitor 
 Returns the elapsed time between the calls of  start()  and
  stop() .
   getDuration()   - 
Method in interface sbrt.kernel.util. ProgressMonitor 
 Returns the current duration of the process being monitored.
   getEdges()   - 
Method in class sbrt.kernel.math.graph_theory. DirectedGraph 
 Returns the set of all edges contained in this graph.
   getEdges(String)   - 
Static method in class sbrt.shell.mng.graph_theory. PathIdManager 
 Parses the file with the provided name, and returns the set of edges it
 contains.
   getEdges(String, DirEdgeFormat<N>)   - 
Static method in class sbrt.shell.mng.graph_theory. PathIdManager 
 Parses the file with the provided name, and returns the set of edges it
 contains.
   getElement(Object, Object)   - 
Method in interface sbrt.kernel.math.algebra. DoubleMatrix 
 Returns the element of this matrix at the provided
 row and column indices.
   getElement(Object, Object)   - 
Method in class sbrt.kernel.math.algebra. DoubleMatrixWrap 
 Returns the element of this matrix at the provided
 row and column indices.
   getElement(Object, Object)   - 
Method in class sbrt.kernel.math.algebra. HashMatrix 
 Returns the element of this matrix at the provided
 row and column indices.
   getElement(Object, Object)   - 
Method in interface sbrt.kernel.math.algebra. Matrix 
 Returns the element of this matrix at the provided
 row and column indices.
   getElement()   - 
Method in class sbrt.kernel.math.algebra. MatrixElement 
 Returns the value of this matrix element.
   getElement(Object, Object)   - 
Method in class sbrt.kernel.math.algebra. MatrixWrap 
 Returns the element of this matrix at the provided
 row and column indices.
   getElement(Object, Object)   - 
Method in class sbrt.kernel.math.algebra. RowOrTreeMatrix 
 Returns the element of this matrix at the provided
 row and column indices.
   getElement(Object, Object)   - 
Method in class sbrt.kernel.math.algebra. TreeMatrix 
 Returns the element of this matrix at the provided
 row and column indices.
   getElementFormat()   - 
Method in interface sbrt.shell.text. CollectionFormat 
 Returns the format object used for 
 the elements of this collection format.
   getElementFormat()   - 
Method in class sbrt.shell.text.math. MatrixElementFormatV1 
 Returns the format used for matrix elements.
   getElementFormatter()   - 
Method in class sbrt.shell.text. AbstractLinkedHashSetFormat 
 Returns the formatter for the elements of this 
  LinkedHashSet  format.
   getElementFormatter()   - 
Method in class sbrt.shell.text. AbstractListFormat 
 Returns the formatter for the elements of this 
  LinkedHashSet  format.
   getElementFormatter()   - 
Method in class sbrt.shell.text. BoolRelFormatV1 
 Returns the formatter for 
 the elements of this set format.
   getElementFormatter()   - 
Method in interface sbrt.shell.text. LinkedHashSetFormat 
 Returns the formatter for 
 the elements of this set format.
   getElementFormatter()   - 
Method in interface sbrt.shell.text. ListFormat 
 Returns the formatter for 
 the elements of this list format.
   getElementFormatter()   - 
Method in class sbrt.shell.text. SimpleListFormat 
 Returns the formatter for 
 the elements of this list format.
   getElementFormatter()   - 
Method in class sbrt.shell.text. SimpleSetFormat 
 Returns the formatter for 
 the elements of this set format.
   getElementParser()   - 
Method in class sbrt.shell.text. AbstractLinkedHashSetFormat 
 Returns the parser for 
 the elements of this set format.
   getElementParser()   - 
Method in class sbrt.shell.text. AbstractListFormat 
 Returns the parser for 
 the elements of this set format.
   getElementParser()   - 
Method in class sbrt.shell.text. BoolRelFormatV1 
 Returns the parser for 
 the elements of this set format.
   getElementParser()   - 
Method in interface sbrt.shell.text. LinkedHashSetFormat 
 Returns the parser for 
 the elements of this set format.
   getElementParser()   - 
Method in interface sbrt.shell.text. ListFormat 
 Returns the parser for 
 the elements of this list format.
   getElementParser()   - 
Method in class sbrt.shell.text. SimpleListFormat 
 Returns the parser for 
 the elements of this list format.
   getElementParser()   - 
Method in class sbrt.shell.text. SimpleSetFormat 
 Returns the parser for 
 the elements of this set format.
   getElements()   - 
Method in class sbrt.kernel.util. BooleanRelation 
  
   getEnzymeChecker()   - 
Method in class sbrt.shell.text.fba. CatalystListFormatV1 
 Returns the catalyst name verifier.
   getEnzymeChecker()   - 
Method in class sbrt.shell.text.fba. CatalystSetFormatV1 
 Returns the catalyst name verifier.
   getEnzymeRxnAssoc()   - 
Method in class sbrt.kernel.fba.proc. NetworkInfoProcess.Results 
 Returns the catalyst-reaction associations contained in the fluxome.
   getEquations(ManagerInput<String, String>, PrintWriter)   - 
Static method in class sbrt.shell.mng.algebra. LinearSolverManager 
 Returns the set of linear equations contained in the file with the
 provided name.
   getEquations(String, PrintWriter)   - 
Static method in class sbrt.shell.mng.algebra. LinearSolverManager 
 Returns the set of linear equations contained in the file with the
 provided name.
   getEquations(String)   - 
Static method in class sbrt.shell.mng.algebra. LinearSolverManager 
 Returns the set of linear equations contained in the file with the
 provided name.
   getEquivRxnNames()   - 
Method in interface sbrt.kernel.fba. Fluxome 
 Returns a collection of sets of the names of stoichiometrically 
 equivalent reactions contained in this fluxome.
   getEquivRxnNames(String)   - 
Method in interface sbrt.kernel.fba. Fluxome 
 Returns the set of names of reactions in this fluxome that are
 stoichiometrically equivalent to the provided reaction.
   getEquivRxnNames()   - 
Method in class sbrt.kernel.fba. IrrevRxnFluxomeV1 
 Returns a collection of sets of the names of stoichiometrically 
 equivalent reactions contained in this fluxome.
   getEquivRxnNames(String)   - 
Method in class sbrt.kernel.fba. IrrevRxnFluxomeV1 
 Returns the set of names of internal reactions in this fluxome that are
 stoichiometrically equivalent to the provided reaction.
   getEquivRxns(String)   - 
Method in interface sbrt.kernel.fba. IrrevRxnFluxome 
 Returns the set reactions in this fluxome that are
 stoichiometrically equivalent to the provided reaction.
   getEquivRxns(String)   - 
Method in class sbrt.kernel.fba. IrrevRxnFluxomeV1 
 Returns the reactions in this fluxome that are
 stoichiometrically equivalent to the provided reaction.
   getEquivRxns()   - 
Method in class sbrt.kernel.fba.proc. NetworkInfoProcess.Results 
 Returns the equivalent reactions contained in the fluxome.
   getExchangeRxn(String)   - 
Method in class sbrt.kernel.fba. IrrevRxnFluxomeV1 
 Returns the exchange reaction in this fluxome with the provided name.
   getExchangeRxnNames()   - 
Method in interface sbrt.kernel.fba. Fluxome 
 Returns the set of names of all exchange reactions contained 
 in this fluxome.
   getExchangeRxnNames()   - 
Method in class sbrt.kernel.fba. IrrevRxnFluxomeV1 
 Returns the set of names of all exchange reactions in this fluxome.
   getExchangeRxns()   - 
Method in interface sbrt.kernel.fba. Fluxome 
 Returns the set of all exchange reactions contained in this fluxome.
   getExchangeRxns()   - 
Method in class sbrt.kernel.fba. IrrevRxnFluxomeV1 
 Returns all of the exchange reactions contained in this fluxome.
   getExpr(V)   - 
Method in class sbrt.kernel.fba.comp. FbaOptComputer 
 Converts a column variable to a single-term linear combination.
   getExpr(Collection<V>)   - 
Method in class sbrt.kernel.fba.comp. FbaOptComputer 
 Converts a collection of column variables to a linear combination.
   getExprConstraints()   - 
Method in class sbrt.kernel.fba. FluxConstraints 
 Returns the constraints for mathematical expressions of fluxes.
   getExpressions(ManagerInput<String, String>, PrintWriter)   - 
Static method in class sbrt.shell.mng.utilities. VariableParticipationManager 
 Opens the file denoted by   VariableParticipationManager.EXPRESSION_FILE   in the 
 provided manager input and returns the list of mathematical expressions
 it contains.
   getFbaOptFileLineFormat()   - 
Method in class sbrt.shell.io.fba. CofoofSource 
 Returns the FBA optimization file line format used to write data strings
 to files produced by this source.
   getFbaOptFileLineFormat()   - 
Method in class sbrt.shell.io.fba. ColumnOrFbaOptOutputFile 
 Returns the FBA optimization file line format used
 to write data strings to this file.
   getFbaOptFileLineFormat()   - 
Method in interface sbrt.shell.io.fba. FbaOptOutputFile 
 Returns the FBA optimization output file line format.
   getFbaOptFileLineFormat()   - 
Method in class sbrt.shell.io.fba. RowOrFbaOptOutputFile 
 Returns the FBA optimization file line format used
 to write data strings to this file.
   getFbaOptFileLineFormat()   - 
Method in class sbrt.shell.io.fba. SerFbaOptOutputFile 
 Returns the FBA optimization file line format used
 to supply a header object for objective values.
   getFileFormat()   - 
Method in class sbrt.shell.io. ComiFileSource 
 Returns the format used to create files of type   ComiFile  .
   getFileFormat()   - 
Method in class sbrt.shell.io. ComoFileSource 
 Returns the format used to create files of type   ComoFile  .
   getFileFormat()   - 
Method in class sbrt.shell.io.fba. CofoofSource 
 Returns the file format used to create files of type
   ColumnOrFbaOptOutputFile  .
   getFileFormat()   - 
Method in class sbrt.shell.io.fba. RofoofSource 
 Returns the file format used to create files of 
 type   RowOrFbaOptOutputFile  .
   getFileFormat()   - 
Method in class sbrt.shell.io.fba. SfoofSource 
 Returns the file format used to create files of 
 type   SerFbaOptOutputFile  .
   getFileFormat()   - 
Method in class sbrt.shell.io. RomiFileSource 
 Returns the format used to create files of type   RomiFile  .
   getFileFormat()   - 
Method in class sbrt.shell.io. RomoFileSource 
 Returns the format used to create files of type   RomoFile  .
   getFileFormat()   - 
Method in class sbrt.shell.io. SerializedInputFile 
 Returns the format of this file.
   getFileFormat()   - 
Method in class sbrt.shell.io. SerializedOutputFile 
 Returns the format of this file.
   getFileFormat()   - 
Method in class sbrt.shell.io. SmoFileSource 
 Returns the format used to create files of type   SmoFile  .
   getFileFormat()   - 
Method in class sbrt.shell.io. TextInputFile 
 Returns the format of this file.
   getFileFormat(ManagerInput<String, String>)   - 
Static method in class sbrt.shell.mng. AbstractProcessManager 
 Returns the parsed value for   AbstractProcessManager.FILE_FORMAT   in the provided input.
   getFileFormat(String)   - 
Static method in class sbrt.shell.mng. AbstractProcessManager 
 Parses and returns the file format corresponding to the provided string.
   getFileNameSource(ManagerInput<String, String>, PrintWriter)   - 
Static method in class sbrt.shell.mng. AbstractProcessManager 
 Returns the parsed value for   AbstractProcessManager.OUTPUT_FILE_NAME_FILE   in the
 provided input and prints a descriptive message to the provided print
 writer.
   getFilterKeys()   - 
Method in class sbrt.shell.io. AbstractMapOutputFile 
 Returns the set of keys whose values will be
 written to this output file when a map is added.
   getFilterKeys()   - 
Method in class sbrt.shell.io.fba. SerFbaOptOutputFile 
 Returns the set of keys whose values will be
 written to this output file when results are added.
   getFilterKeys()   - 
Method in interface sbrt.shell.io. MapOutputFile 
 Returns the set of keys whose values will be
 written to this output file when a map is added.
   getFilterKeys()   - 
Method in class sbrt.shell.io. SmoFile 
 Returns the set of keys whose values will be
 written to this output file when a map is added.
   getFluxBounds(Node)   - 
Method in class sbrt.shell.text.fba. BiggRxnNodeParser 
 Returns the flux bounds of the provided reaction node.
   getFluxBounds(Node)   - 
Method in class sbrt.shell.text.fba. PalssonRxnNodeParser 
 Returns the flux bounds of the provided reaction node.
   getFluxCap()   - 
Method in exception sbrt.kernel.fba.comp. FluxCapException 
 Returns the invalid flux cap.
   getFluxCaps()   - 
Method in class sbrt.kernel.fba.comp. FluxCapComputer 
 Computes and returns all flux caps.
   getFluxCaps()   - 
Method in class sbrt.shell.io.fba. FluxCapInputFile 
 Returns the flux caps contained in this file.
   getFluxCaps(ManagerInput<String, String>, PrintWriter, Fluxome)   - 
Static method in class sbrt.shell.mng.fba. MsFluxIntervalManager 
 Returns the flux caps contained in the file with the name specified by
   MsFluxIntervalManager.FLUX_CAP_FILE   in the provided input.
   getFluxConstraint(String)   - 
Method in class sbrt.kernel.fba.comp. AbstractFluxIntervalComputer 
 Returns the current flux constraint on the reaction with the provided
 name.
   getFluxConstraint(String)   - 
Method in class sbrt.kernel.fba.comp. CdHarFluxVectorComputer 
 Returns the current flux constraint on the reaction with the provided
 name.
   getFluxConstraint(String)   - 
Method in class sbrt.kernel.fba.comp. FastMsFluxRangeComputer 
 Returns the current flux constraint on the reaction with the provided
 name.
   getFluxConstraint(String)   - 
Method in class sbrt.kernel.fba.comp. FbaOptComputer 
 Returns the current flux constraint on the reaction with the provided
 name.
   getFluxConstraint(String)   - 
Method in interface sbrt.kernel.fba.comp. FluxCvComputer 
 Returns the current flux constraint on the reaction with the provided
 name.
   getFluxConstraint(String)   - 
Method in class sbrt.kernel.fba.comp. InitialPointComputer 
 Returns the current flux constraint on the reaction with the provided
 name.
   getFluxConstraint(String)   - 
Method in class sbrt.kernel.fba.comp. RxnDeletionComputer 
 Returns the flux constraint of the reaction with the provided name.
   getFluxConstraint(String)   - 
Method in interface sbrt.kernel.fba. Fluxome 
 Returns the flux constraint corresponding to the provided
 reaction.
   getFluxConstraint(String)   - 
Method in class sbrt.kernel.fba. IrrevRxnFluxomeV1 
 Returns the flux constraint for the provided reaction.
   getFluxConstraints()   - 
Method in class sbrt.kernel.fba.comp. AbstractFluxIntervalComputer 
 Returns all current flux constraints.
   getFluxConstraints()   - 
Method in class sbrt.kernel.fba.comp. CdHarFluxVectorComputer 
 Returns all current flux constraints.
   getFluxConstraints()   - 
Method in class sbrt.kernel.fba.comp. FastMsFluxRangeComputer 
 Returns all current flux constraints.
   getFluxConstraints()   - 
Method in class sbrt.kernel.fba.comp. FbaOptComputer 
 Returns all current flux constraints for each reaction
 in the fluxome used by this optimization computer.
   getFluxConstraints()   - 
Method in interface sbrt.kernel.fba.comp. FluxCvComputer 
 Returns all current flux constraints.
   getFluxConstraints()   - 
Method in class sbrt.kernel.fba.comp. InitialPointComputer 
 Returns all current flux constraints.
   getFluxConstraints()   - 
Method in class sbrt.kernel.fba.comp. RxnDeletionComputer 
 Returns the set of all constraints on single fluxes.
   getFluxConstraints()   - 
Method in class sbrt.kernel.fba. FluxConstraints 
 Returns the flux constraints for single reactions.
   getFluxConstraints()   - 
Method in class sbrt.kernel.fba. IrrevRxnFluxomeV1 
 Returns the constraints on each flux in this fluxome.
   getFluxIntervalFile(ManagerInput<String, String>, PrintWriter, Fluxome)   - 
Static method in class sbrt.shell.mng.fba. FluxPlasticityManager 
 Returns the flux interval input with the name denoted by
   FluxPlasticityManager.FLUX_INTERVAL_FILE   in the provided manager input.
   getFluxIntervalFile(String, Fluxome)   - 
Static method in class sbrt.shell.mng.fba. FluxPlasticityManager 
 Returns the flux interval input file with the provided name.
   getFluxMax(String)   - 
Method in class sbrt.kernel.fba.comp. FbaOptComputer 
 Computes the maximum flux of the reaction with the provided name.
   getFluxMaxima(Collection<String>)   - 
Method in class sbrt.kernel.fba.comp. FbaOptComputer 
 Computes the maximum flux of each reaction with the provided names.
   getFluxMaxima(Collection<String>, MutableProgressMonitor)   - 
Method in class sbrt.kernel.fba.comp. ReverseRxnFluxIntervalComputer 
 Computes and returns the maximum fluxes of the reactions with the
 provided names.
   getFluxMin(String)   - 
Method in class sbrt.kernel.fba.comp. FbaOptComputer 
 Computes the minimum flux of the reaction with the provided name.
   getFluxMinima(Collection<String>)   - 
Method in class sbrt.kernel.fba.comp. FbaOptComputer 
 Computes the minimum flux of each reaction with the provided names.
   getFluxMinima(Collection<String>, MutableProgressMonitor)   - 
Method in class sbrt.kernel.fba.comp. ReverseRxnFluxIntervalComputer 
 Computes and returns the minimum fluxes of the reactions with the
 provided names.
   getFluxome()   - 
Method in class sbrt.kernel.fba.comp. AbstractCvComputer 
 Returns the fluxome for which values will be computed.
   getFluxome()   - 
Method in class sbrt.kernel.fba.comp. FbaOptComputer 
 Returns the fluxome used by this optimization computer.
   getFluxome()   - 
Method in class sbrt.kernel.fba.comp. FluxCapComputer 
 Returns the fluxome for which flux caps are computed.
   getFluxome()   - 
Method in interface sbrt.kernel.fba.comp. FluxCvComputer 
 Returns the fluxome used to construct this flux constraint-variation
 computer.
   getFluxome()   - 
Method in class sbrt.kernel.fba.comp. InitialPointComputer 
 Returns the fluxome used by this computer.
   getFluxome()   - 
Method in class sbrt.kernel.fba.proc. CatalystDeletionProcess 
 Returns the fluxome used by the reaction deletion computer.
   getFluxome()   - 
Method in class sbrt.kernel.fba.proc. CdHarFluxVectorProcess 
 Returns the fluxome for which flux vectors are to be generated.
   getFluxome()   - 
Method in class sbrt.kernel.fba.proc. CvCatalystDeletionProcess 
 Returns the fluxome used by the reaction deletion computer.
   getFluxome()   - 
Method in class sbrt.kernel.fba.proc. CvFbaOptimizationExperiment 
 Returns the fluxome used in this experiment.
   getFluxome()   - 
Method in class sbrt.kernel.fba.proc. CvFluxIntervalProcess 
 Returns the fluxome used in this experiment.
   getFluxome()   - 
Method in class sbrt.kernel.fba.proc. CvObjectiveFunctionProcess 
 Returns the fluxome used in this experiment.
   getFluxome()   - 
Method in class sbrt.kernel.fba.proc. CvRxnDeletionProcess 
 Returns the fluxome used by the reaction deletion computer.
   getFluxome()   - 
Method in class sbrt.kernel.fba.proc. CycleIdProcess 
 Returns the fluxome used in this process.
   getFluxome()   - 
Method in class sbrt.kernel.fba.proc. ExtremeCurrentProcess 
 Returns the fluxome used in this experiment.
   getFluxome()   - 
Method in class sbrt.kernel.fba.proc. FbaOptimizationProcess 
 Returns the fluxome used in this experiment.
   getFluxome()   - 
Method in class sbrt.kernel.fba.proc. FbaProcess 
 Returns the fluxome used in this experiment.
   getFluxome()   - 
Method in class sbrt.kernel.fba.proc. FbaSolverProcess 
 Returns the fluxome for which Sv = 0 is solved.
   getFluxome()   - 
Method in class sbrt.kernel.fba.proc. FluxActivityProcess 
 Returns the fluxome used in this flux activity experiment.
   getFluxome()   - 
Method in class sbrt.kernel.fba.proc. FluxCapProcess 
 Returns the fluxome for which flux caps are computed.
   getFluxome()   - 
Method in class sbrt.kernel.fba.proc. FluxIntervalProcess 
 Returns the fluxome used in this experiment.
   getFluxome()   - 
Method in class sbrt.kernel.fba.proc. FluxomeReducerProcess 
 Returns the fluxome used in this process.
   getFluxome()   - 
Method in class sbrt.kernel.fba.proc. FluxPlasticityProcess 
 Returns the fluxome used in this flux plasticity experiment.
   getFluxome()   - 
Method in class sbrt.kernel.fba.proc. InitialPointGenerator 
 Returns the fluxome used in this initial point experiment.
   getFluxome()   - 
Method in class sbrt.kernel.fba.proc. NetworkInfoProcess 
 Returns the fluxome for which information is to be gathered.
   getFluxome()   - 
Method in class sbrt.kernel.fba.proc. ObjectiveFunctionAnalysis 
 Returns the fluxome used in this experiment.
   getFluxome()   - 
Method in class sbrt.kernel.fba.proc. RandomConstraintsGeneratorV1 
 Returns the fluxome for which random objective functions are to be
 generated.
   getFluxome()   - 
Method in class sbrt.kernel.fba.proc. RandomEnvGeneratorV1 
 Returns the fluxome for which random objective functions are to be
 generated.
   getFluxome()   - 
Method in class sbrt.kernel.fba.proc. RandomObjectiveGenerator 
 Returns the fluxome for which random objective functions are to be
 generated.
   getFluxome()   - 
Method in class sbrt.kernel.fba.proc. RxnDeletionProcess 
 Returns the fluxome used by the reaction deletion computer.
   getFluxome()   - 
Method in class sbrt.kernel.fba.proc. StoichVariationProcess 
 Returns the fluxome used in this process.
   getFluxome()   - 
Method in class sbrt.kernel.fba.proc. SvCdHarFluxVectorProcess 
 Returns the fluxome for which flux vectors are to be generated.
   getFluxome()   - 
Method in class sbrt.kernel.fba.proc. SvInitialPointProcess 
 Returns the fluxome used in this initial point experiment.
   getFluxome()   - 
Method in class sbrt.shell.io.fba. FluxCapInputFile 
 Returns the fluxome used to verify reaction names.
   getFluxome(ManagerInput<String, String>, PrintWriter)   - 
Static method in class sbrt.shell.mng.fba. FbaManager 
 Opens the file denoted by   FbaManager.RXN_FILE   in the 
 provided manager input and returns the fluxome it contains.
   getFluxome(String, PrintWriter)   - 
Static method in class sbrt.shell.mng.fba. FbaManager 
 Returns the fluxome contained in the file with the provided name.
   getFluxome(String)   - 
Static method in class sbrt.shell.mng.fba. FbaManager 
 Returns the fluxome contained in the file with the provided name.
   getFluxome(String, String)   - 
Static method in class sbrt.shell.mng.fba. FbaManager 
 Returns the fluxome contained in the files with the provided names.
   getFluxome(String, String, PrintWriter)   - 
Static method in class sbrt.shell.mng.fba. FbaManager 
 Returns the fluxome contained in the files with the provided names.
   getFluxome(ManagerInput<String, String>, PrintWriter)   - 
Static method in class sbrt.shell.mng.fba. FbaOptManager 
 Creates and returns a fluxome based on the provided input.
   getFluxome(ManagerInput<String, String>, PrintWriter, double)   - 
Static method in class sbrt.shell.mng.fba. FbaOptManager 
 Creates and returns a fluxome based on the provided input.
   getFluxome(ManagerInput<String, String>, PrintWriter, double)   - 
Static method in class sbrt.shell.mng.fba. InitialPointManager 
 Creates and returns a fluxome based on the provided input.
   getFluxome(ManagerInput<String, String>, PrintWriter)   - 
Static method in class sbrt.shell.mng.fba. RandomFluxVectorManager 
 Creates and returns a fluxome based on the provided input.
   getFluxome()   - 
Method in interface sbrt.shell.text.fba. CatalystListFormat 
 Returns the fluxome used to verify catalyst names.
   getFluxome()   - 
Method in class sbrt.shell.text.fba. CatalystListFormatV1 
 Returns the fluxome used to verify catalyst names.
   getFluxome()   - 
Method in interface sbrt.shell.text.fba. CatalystSetFormat 
 Returns the fluxome used to verify enzyme names.
   getFluxome()   - 
Method in class sbrt.shell.text.fba. CatalystSetFormatV1 
 Returns the fluxome used to verify catalyst names.
   getFluxome()   - 
Method in class sbrt.shell.text.fba. CatalystVerifier 
 Returns the fluxome used to create this catalyst name verifier.
   getFluxome()   - 
Method in class sbrt.shell.text.fba. ChemSpeciesVerifier 
 Returns the fluxome used to create this chemical species verifier.
   getFluxome()   - 
Method in interface sbrt.shell.text.fba. ConstraintsFileLineFormat 
 Returns the fluxome used to verify reaction names.
   getFluxome()   - 
Method in class sbrt.shell.text.fba. ConstraintsFileLineFormatV1 
 Returns the fluxome used to verify reaction names.
   getFluxome()   - 
Method in class sbrt.shell.text.fba. ConstraintsFileLineFormatV2 
 Returns the fluxome used to verify reaction names.
   getFluxome()   - 
Method in interface sbrt.shell.text.fba. FbaOptFileLineFormat 
 Returns the fluxome used to verify reaction names.
   getFluxome()   - 
Method in class sbrt.shell.text.fba. FbaOptFileLineFormatV1 
 Returns the fluxome used to verify reaction names.
   getFluxome()   - 
Method in interface sbrt.shell.text.fba. FbaOptHeaderFormatter 
 Returns the fluxome used to verify reaction names.
   getFluxome()   - 
Method in class sbrt.shell.text.fba. FbaOptHeaderFormatterV1 
 Returns the fluxome used to verify reaction names.
   getFluxome()   - 
Method in interface sbrt.shell.text.fba. FbaOptHeaderParser 
 Returns the fluxome used to verify reaction names.
   getFluxome()   - 
Method in class sbrt.shell.text.fba. FbaOptHeaderParserV1 
 Returns the fluxome used to verify reaction names.
   getFluxome()   - 
Method in interface sbrt.shell.text.fba. FluxCapFormat 
 Returns the fluxome used to verify reaction names.
   getFluxome()   - 
Method in class sbrt.shell.text.fba. FluxCapFormatV1 
 Returns the fluxome used to verify reaction names.
   getFluxome()   - 
Method in class sbrt.shell.text.fba. FluxomeSolFileLineFormatV1 
 Returns the fluxome used to verify reaction names.
   getFluxome()   - 
Method in interface sbrt.shell.text.fba. FluxomeSolutionFileLineFormat 
 Returns the fluxome used to verify reaction names.
   getFluxome()   - 
Method in interface sbrt.shell.text.fba. FluxVectorFormat 
 Returns the fluxome used to verify reaction names.
   getFluxome()   - 
Method in class sbrt.shell.text.fba. FluxVectorFormatV1 
 Returns the fluxome used to verify reaction names.
   getFluxome()   - 
Method in interface sbrt.shell.text.fba. RxnNameExprFormat 
 Returns the fluxome used to verify reaction names.
   getFluxome()   - 
Method in class sbrt.shell.text.fba. RxnNameExprFormatV1 
 Returns the fluxome used to verify reaction names.
   getFluxome()   - 
Method in interface sbrt.shell.text.fba. RxnNameListFormat 
 Returns the fluxome used to verify reaction names.
   getFluxome()   - 
Method in class sbrt.shell.text.fba. RxnNameListFormatV1 
 Returns the fluxome used to verify reaction names.
   getFluxome()   - 
Method in interface sbrt.shell.text.fba. RxnNameOrExprFormat 
 Returns the fluxome used to verify reaction names.
   getFluxome()   - 
Method in class sbrt.shell.text.fba. RxnNameOrExprFormatV1 
 Returns the fluxome used to verify reaction names.
   getFluxome()   - 
Method in interface sbrt.shell.text.fba. RxnNameSetFormat 
 Returns the fluxome used to verify reaction names.
   getFluxome()   - 
Method in class sbrt.shell.text.fba. RxnNameSetFormatV1 
 Returns the fluxome used to verify reaction names.
   getFluxome()   - 
Method in class sbrt.shell.text.fba. RxnNameVerifier 
 Returns the fluxome used to create this reaction name
 checker.
   getFluxomeSolution(ManagerInput<String, String>, PrintWriter, Fluxome)   - 
Static method in class sbrt.shell.mng.fba. FbaManager 
 Opens the file denoted by   FbaManager.SYSTEM_SOLUTION_FILE   in the 
 provided manager input and returns the linear system solution 
 it contains.
   getFluxValue(Node)   - 
Method in class sbrt.shell.text.fba. BiggRxnNodeParser 
 Returns the flux value of the provided reaction node.
   getFluxValue(Node)   - 
Method in class sbrt.shell.text.fba. PalssonRxnNodeParser 
 Returns the flux value of the provided reaction node.
   getFluxVector()   - 
Method in class sbrt.kernel.fba.comp. CdHarFluxVectorComputer.Results 
 Returns the random interior flux vector that was computed.
   getFluxVector()   - 
Method in class sbrt.kernel.fba.comp. FbaOptComputer.Results 
 Returns the flux vector computed during optimization.
   getFluxVector()   - 
Method in class sbrt.kernel.fba.comp. InitialPointComputer.Results 
 Returns the computed flux vector.
   getFluxVector()   - 
Method in exception sbrt.kernel.fba.comp. InvalidFluxVectorException 
 Returns the invalid flux vector;
   getFluxVector(ManagerInput<String, String>, PrintWriter, Fluxome, double)   - 
Static method in class sbrt.shell.mng.fba. CdHarFluxVectorManager 
 Opens the file denoted by   CdHarManager.INITIAL_POINT_FILE   in the
 provided manager input and returns the flux vector it contains.
   getFluxVector(ManagerInput<String, String>, PrintWriter, Fluxome, double)   - 
Static method in class sbrt.shell.mng.fba. RandomFluxVectorManager 
 Opens the file denoted by   FbaManager.FLUX_VECTOR_FILE   in the 
 provided manager input and returns the flux vector it contains.
   getFluxVector(String, Fluxome, double)   - 
Static method in class sbrt.shell.mng.fba. RandomFluxVectorManager 
 Returns the flux vector contained in the file with the provided name.
   getFluxVectorFile(ManagerInput<String, String>, PrintWriter, Fluxome)   - 
Static method in class sbrt.shell.mng.fba. FluxActivityManager 
 Returns the flux vector input file specified by the provided input.
   getFluxVectorFile(String, FileFormat, Fluxome)   - 
Static method in class sbrt.shell.mng.fba. FluxActivityManager 
 Returns the flux vector input file with the provided name.
   getFluxVectorFile(ManagerInput<String, String>, PrintWriter, Fluxome)   - 
Static method in class sbrt.shell.mng.fba. InitialPointManager 
 Returns an empty output file that is suitable for storing a single flux
 vector.
   getFluxVectorFile(ManagerInput<String, String>, PrintWriter, Fluxome)   - 
Static method in class sbrt.shell.mng.fba. SvInitialPointManager 
 Returns an empty output file that is suitable for storing multiple flux
 vectors.
   getFreeVariables()   - 
Method in class sbrt.kernel.math.algebra. HashLinearSystemSolution 
 Returns the set of free variables contained in this solution.
   getFreeVariables()   - 
Method in interface sbrt.kernel.math.algebra. LinearSystemSolution 
 Returns the set of free variables contained in this
 solution.
   getGenes(Node)   - 
Method in class sbrt.shell.text.fba. BiggRxnNodeParser 
 Calls  BiggRxnNodeParser.getCatalysts(Node, String)  with " GENE ASSOCIATION: "
 as the  prefix .
   getGenes(Node)   - 
Method in class sbrt.shell.text.fba. PalssonRxnNodeParser 
 Calls  PalssonRxnNodeParser.getCatalysts(Node, String)  with " GENE_ASSOCIATION: "
 as the  prefix .
   getGraph()   - 
Method in class sbrt.kernel.math.graph_theory.comp. PathFinder 
 Returns the graph in which pathways will be computed.
   getGraph(ManagerInput<String, String>, PrintWriter)   - 
Static method in class sbrt.shell.mng.graph_theory. PathIdManager 
 Opens the file denoted by   PathIdManager.EDGE_FILE   in the provided manager
 input and returns the directed graph it contains.
   getGraph(String)   - 
Static method in class sbrt.shell.mng.graph_theory. PathIdManager 
 Parses the file with the provided name, and returns the directed graph it
 contains.
   getGraph(String, DirEdgeFormat<N>)   - 
Static method in class sbrt.shell.mng.graph_theory. PathIdManager 
 Parses the file with the provided name, and returns the directed graph it
 contains.
   getInactive()   - 
Method in class sbrt.kernel.fba.proc. RandomConstraintsGeneratorV1 
 Returns the constraint used to represent an inactive flux.
   getInactive()   - 
Method in class sbrt.kernel.fba.proc. RandomEnvGeneratorV1 
 Returns the constraint used to represent an inactive source flux.
   getInactive(ManagerInput<String, String>)   - 
Static method in class sbrt.shell.mng.fba. RandomConstraintsManager 
 Parses and returns the value for   RandomConstraintsManager.INACTIVE   in the 
 provided manager input.
   getIndexFormat()   - 
Method in class sbrt.shell.text.math. MatrixElementFormatV1 
 Returns the format used for matrix elements.
   getIndices()   - 
Method in class sbrt.kernel.math.algebra. MatrixElement 
 Returns the pair of indices.
   getInitialFluxVectorFile(ManagerInput<String, String>, PrintWriter, Fluxome)   - 
Static method in class sbrt.shell.mng.fba. SvCdHarFluxVectorManager 
 Returns the input file containing a collection of initial flux vectors.
   getInitialPoint(ManagerInput<String, String>, PrintWriter, Set<String>)   - 
Static method in class sbrt.shell.mng.geom. CdHarManager 
 Opens the file with the name denoted by   CdHarManager.INITIAL_POINT_FILE   and
 return the vector it contains.
   getInitialPoint(String, PrintWriter, Set<String>)   - 
Static method in class sbrt.shell.mng.geom. CdHarManager 
 Opens the file with the provided name and return the vector it contains.
   getInitialPoint(String, Set<String>)   - 
Static method in class sbrt.shell.mng.geom. CdHarManager 
 Opens the file with the provided name and return the vector it contains.
   getInitialPoint(String, MapFormat<String, Double>, Set<String>)   - 
Static method in class sbrt.shell.mng.geom. CdHarManager 
 Opens the file with the provided name and return the vector it contains.
   getInputFile(ManagerInput<String, String>, PrintWriter, Fluxome)   - 
Static method in class sbrt.shell.mng.fba. ObjectiveFunctionManager 
 Returns the file denoted by   ObjectiveFunctionManager.OBJ_FUNCT_FILE   in the 
 provided manager input.
   getInputFile(String, FileFormat, Fluxome)   - 
Static method in class sbrt.shell.mng.fba. ObjectiveFunctionManager 
 Returns the input file with the provided
 name.
   getInputFile(String, Fluxome)   - 
Static method in class sbrt.shell.mng.fba. ObjectiveFunctionManager 
 Returns the input file with the provided
 name.
   getInputFile(ManagerInput<String, String>, PrintWriter, Fluxome)   - 
Static method in class sbrt.shell.mng.fba. RxnDeletionManager 
 Parses and returns the value for   RxnDeletionManager.RXN_DELETION_FILE   
 in the provided manager input.
   getInputFile(String, Fluxome)   - 
Static method in class sbrt.shell.mng.fba. RxnDeletionManager 
 Returns the reaction name input file with the provided name.
   getInputIrrevRxnFormat()   - 
Method in class sbrt.shell.proc.fba. SimpleRxnFileTranslator 
 Returns the parser used for irreversible reactions in the
 input file.
   getInputRevRxnFormat()   - 
Method in class sbrt.shell.proc.fba. SimpleRxnFileTranslator 
 Returns the parser used for reversible reactions in the
 input file.
   getInternalRxn(String)   - 
Method in interface sbrt.kernel.fba. IrrevRxnFluxome 
 Returns the internal reaction in this fluxome with the provided name.
   getInternalRxn(String)   - 
Method in class sbrt.kernel.fba. IrrevRxnFluxomeV1 
 Returns the internal reaction in this fluxome with the provided name.
   getInternalRxnNames()   - 
Method in interface sbrt.kernel.fba. Fluxome 
 Returns the set of names of all internal chemical reactions
 contained in this fluxome.
   getInternalRxnNames()   - 
Method in class sbrt.kernel.fba. IrrevRxnFluxomeV1 
 Returns the set of names of all internal reactions in this fluxome.
   getInternalRxns()   - 
Method in interface sbrt.kernel.fba. Fluxome 
 Returns the set of all internal chemical reactions contained
 in this fluxome.
   getInternalRxns()   - 
Method in interface sbrt.kernel.fba. IrrevRxnFluxome 
 Returns the set of all internal reactions in this fluxome.
   getInternalRxns(Collection<String>)   - 
Method in interface sbrt.kernel.fba. IrrevRxnFluxome 
 Returns the internal reactions with the provided names.
   getInternalRxns()   - 
Method in class sbrt.kernel.fba. IrrevRxnFluxomeV1 
 Returns all of the internal reactions in this fluxome.
   getInternalRxns(Collection<String>)   - 
Method in class sbrt.kernel.fba. IrrevRxnFluxomeV1 
 Returns the internal reactions whose names appear in the provided
 collection of reaction names.
   getInterval(String, Interval, double, double)   - 
Method in class sbrt.kernel.fba.comp. AbstractFluxIntervalComputer 
 Returns the provided minimum and maximum fluxes as an interval.
   getInterval1()   - 
Method in class sbrt.kernel.math. IntervalComparer.Diff 
  
   getInterval2()   - 
Method in class sbrt.kernel.math. IntervalComparer.Diff 
  
   getIntervalFormat()   - 
Method in class sbrt.shell.text.math. BoundedIntervalFormatV1 
 Returns the interval format used by this format.
   getIntervalInputFile(String, Fluxome, MapFormat<LinearComb<String>, Interval>)   - 
Static method in class sbrt.shell.mng.fba. IntervalVariationManager 
 Returns the flux interval input file with the provided name.
   getIntervalInputFile(String, Fluxome)   - 
Static method in class sbrt.shell.mng.fba. IntervalVariationManager 
 Returns the flux interval input file with the provided name.
   getIntervals(Map<String, Double>, Map<String, Double>)   - 
Method in class sbrt.kernel.fba.comp. AbstractFluxIntervalComputer 
 Returns the provided minima and maxima as intervals.
   getIntervals()   - 
Method in class sbrt.kernel.fba.comp. FastMsFluxRangeComputer 
 Computes and returns the intervals of every flux in the fluxome
 used to construct this flux interval computer.
   getIntervals(Collection<String>)   - 
Method in class sbrt.kernel.fba.comp. FastMsFluxRangeComputer 
 Computes and returns the flux intervals of the reactions with the
 provided names.
   getIntervals()   - 
Method in interface sbrt.kernel.fba.comp. FluxIntervalComputer 
 Computes and returns the intervals of every flux in the fluxome
 used to construct this flux interval computer.
   getIntervals(Collection<String>)   - 
Method in interface sbrt.kernel.fba.comp. FluxIntervalComputer 
 Computes and returns the flux intervals of the reactions with the
 provided names.
   getIntervals()   - 
Method in class sbrt.kernel.fba.comp. MsFluxIntervalComputer 
 Computes and returns the intervals of every flux in the fluxome
 used to construct this flux interval computer.
   getIntervals(Collection<String>)   - 
Method in class sbrt.kernel.fba.comp. MsFluxIntervalComputer 
 Computes and returns the flux intervals of the reactions with the
 provided names.
   getIntervals()   - 
Method in class sbrt.kernel.fba.comp. ReverseRxnFluxIntervalComputer 
 Computes and returns the intervals of every flux in the fluxome used to
 construct this flux interval computer.
   getIntervals(Collection<String>)   - 
Method in class sbrt.kernel.fba.comp. ReverseRxnFluxIntervalComputer 
 Computes and returns the flux intervals of the reactions with the
 provided names.
   getIntervals()   - 
Method in class sbrt.kernel.fba.comp. SimpleFluxIntervalComputer 
 Computes and returns the intervals of every flux in the fluxome used to
 construct this flux interval computer.
   getIntervals(Collection<String>)   - 
Method in class sbrt.kernel.fba.comp. SimpleFluxIntervalComputer 
 Computes and returns the flux intervals of the reactions with the
 provided names.
   getIntervals(ManagerInput<String, String>, PrintWriter, Fluxome)   - 
Static method in class sbrt.shell.mng.fba. InitialPointManager 
 Opens the file denoted by   InitialPointManager.FLUX_INTERVAL_FILE   in the provided
 manager input and returns the set of flux intervals it contains.
   getIntervals(String, Fluxome)   - 
Static method in class sbrt.shell.mng.fba. InitialPointManager 
 Returns the set of flux intervals contained in the file with the provided
 name.
   getIterations()   - 
Method in class sbrt.kernel.fba.proc. CdHarFluxVectorProcess 
 Returns the total number of flux vectors to be generated.
   getIterations()   - 
Method in class sbrt.kernel.fba.proc. RandomConstraintsGeneratorV1 
 Returns the number of sets of random constraints to be generated.
   getIterations()   - 
Method in class sbrt.kernel.fba.proc. RandomEnvGeneratorV1 
 Returns the number of random environments to be generated.
   getIterations(ManagerInput<String, String>, PrintWriter)   - 
Static method in class sbrt.shell.mng. AbstractProcessManager 
 Returns the parsed value for   AbstractProcessManager.ITERATIONS   in the provided input
 and prints a descriptive message to the provided print writer.
   getKeyFormat()   - 
Method in class sbrt.shell.text. ColonMapEntryFormatV1 
 Returns the format used for keys.
   getKeyFormat()   - 
Method in class sbrt.shell.text. EqualsMapEntryFormatV1 
 Returns the format used for keys.
   getKeyFormatter()   - 
Method in class sbrt.shell.text. AbstractMapFormat 
 Returns the formatter used for keys.
   getKeyParser()   - 
Method in class sbrt.shell.text. AbstractMapFormat 
 Returns the parser used for keys.
   getKeys()   - 
Method in class sbrt.shell.io. ComiFile 
 Returns the map keys (column headers) contained in this file.
   getKeys()   - 
Method in class sbrt.shell.io. RomiFile 
 Returns the keys of the map contained in this file.
   getLargest(double, double)   - 
Static method in class sbrt.kernel.util. Util 
 Returns the largest of the provided numbers.
   getLargest(int, int)   - 
Static method in class sbrt.kernel.util. Util 
 Returns the largest of the provided numbers.
   getLeftPadding()   - 
Method in class sbrt.shell.text. AbstractMapEntryFormat 
 Returns the left padding used to create formatted
 string representations of key-value pairs.
   getLinearCombFormat()   - 
Method in class sbrt.shell.text.chem. IrrevRxnFormatV1 
 Returns the format used for stoichiometric expressions.
   getLinearCombFormat()   - 
Method in class sbrt.shell.text.chem. IrrevRxnFormatV2 
 Returns the format used for stoichiometric expressions.
   getLinearCombFormat()   - 
Method in class sbrt.shell.text.chem. RevRxnFormatV1 
 Returns the format used for stoichiometric expressions.
   getLinearCombFormat()   - 
Method in class sbrt.shell.text.chem. RevRxnFormatV2 
 Returns the format used for stoichiometric expressions.
   getLinearCombFormat()   - 
Method in class sbrt.shell.text.fba. IrrevRxnFormatV1 
 Returns the format used for stoichiometric expressions.
   getLinearCombFormat()   - 
Method in class sbrt.shell.text.fba. RxnNameOrExprFormatV1 
 Returns the reaction name expression format.
   getLineFormat()   - 
Method in class sbrt.shell.io. ComiFileSource 
 Returns the map format used to create files of type   ComiFile  .
   getLineFormat()   - 
Method in class sbrt.shell.io. ComoFileSource 
 Returns the map format used to create files of type   ComoFile  .
   getLineFormat()   - 
Method in class sbrt.shell.io. RomiFileSource 
 Returns the map format used to create files of type   RomiFile  .
   getLineFormat()   - 
Method in class sbrt.shell.io. RomoFileSource 
 Returns the map format used to create files of type   RomoFile  .
   getLineFormat()   - 
Method in class sbrt.shell.io. TextInputFile 
 Returns the format used to parse lines of this file.
   getLineFormat()   - 
Method in class sbrt.shell.io. TextOutputFile 
 Returns the format used to write lines to this
 file.
   getLineFormat()   - 
Method in class sbrt.shell.proc.fba. SimpleRxnFileTranslator 
 Returns the format used for individual lines in the input and 
 output files.
   getLinerCombFormat()   - 
Method in class sbrt.shell.text.fba. RxnNameExprFormatV1 
 Returns the format used for linear combination.
   getList()   - 
Static method in class sbrt.shell.text. SpecialChars 
 Returns a list of the characters contained in this class.
   getListFormat()   - 
Method in class sbrt.shell.text. AbstractMapFormat 
 Returns the format used for lists of values.
   getListFormat()   - 
Method in class sbrt.shell.text. AppInputFileLineFormatVersion1 
 Returns the format used for lists of values.
   getListFormat()   - 
Method in class sbrt.shell.text. ColonMapFormatV1 
 Returns the format used for lists of values.
   getListFormat()   - 
Method in class sbrt.shell.text. EqualsMapFormatV1 
 Returns the format used for lists of values.
   getListFormat()   - 
Method in class sbrt.shell.text.fba. CatalystListFormatV1 
 Returns the format used for lists of catalyst names.
   getListFormat()   - 
Method in class sbrt.shell.text.fba. ConstraintsFileLineFormatV1 
 Returns the format used for lists of values.
   getListFormat()   - 
Method in class sbrt.shell.text.fba. ConstraintsFileLineFormatV2 
 Returns the format used for lists of values.
   getListFormat()   - 
Method in class sbrt.shell.text.fba. FluxCapFormatV1 
 Returns the format used for lists of values.
   getListFormat()   - 
Method in class sbrt.shell.text.fba. FluxomeSolFileLineFormatV1 
 Returns the format used for lists of values.
   getListFormat()   - 
Method in class sbrt.shell.text.fba. FluxVectorFormatV1 
 Returns the format used for lists of values.
   getListFormat()   - 
Method in class sbrt.shell.text.fba. IrfFormatV1 
 Returns the format used for lists of reactions.
   getListFormat()   - 
Method in class sbrt.shell.text.fba. RxnNameListFormatV1 
 Returns the format used for lists of reaction names.
   getListFormat()   - 
Method in class sbrt.shell.text. SimpleListFormat 
 Returns the underlying list format.
   getLower()   - 
Method in exception sbrt.kernel.math. IllegalBoundsException 
 Returns the lower bound used to create this exception.
   getLower()   - 
Method in class sbrt.kernel.math. Interval 
 Returns the lower bound (or endpoint) of this interval.
   getLowerBound(Node)   - 
Method in class sbrt.shell.text.fba. BiggRxnNodeParser 
 Returns the lower flux bound of the provided reaction node.
   getLowerBound(Node)   - 
Method in class sbrt.shell.text.fba. PalssonRxnNodeParser 
 Returns the lower flux bound of the provided reaction node.
   getMap()   - 
Method in class sbrt.shell.io. RomiFile 
 Returns the map contained in this file.
   getMapEntryFormat()   - 
Method in class sbrt.shell.text. AbstractMapFormat 
 Returns the format used for variable-value pairs.
   getMapEntryFormat()   - 
Method in class sbrt.shell.text. AppInputFileLineFormatVersion1 
 Returns the format used for variable-value pairs.
   getMapEntryFormat()   - 
Method in class sbrt.shell.text. ColonMapFormatV1 
 Returns the format used for variable-value pairs.
   getMapEntryFormat()   - 
Method in class sbrt.shell.text. EqualsMapFormatV1 
 Returns the format used for variable-value pairs.
   getMapEntryFormat()   - 
Method in class sbrt.shell.text.fba. ConstraintsFileLineFormatV1 
 Returns the format used for variable-value pairs.
   getMapEntryFormat()   - 
Method in class sbrt.shell.text.fba. ConstraintsFileLineFormatV2 
 Returns the format used for variable-value pairs.
   getMapEntryFormat()   - 
Method in class sbrt.shell.text.fba. FluxCapFormatV1 
 Returns the format used for variable-value pairs.
   getMapEntryFormat()   - 
Method in class sbrt.shell.text.fba. FluxomeSolFileLineFormatV1 
 Returns the format used for variable-value pairs.
   getMapEntryFormat()   - 
Method in class sbrt.shell.text.fba. FluxVectorFormatV1 
 Returns the format used for variable-value pairs.
   getMapEntryFormat()   - 
Method in class sbrt.shell.text.fba. IrfFormatV1 
 Returns the format used for reaction name-reaction pairs.
   getMapFormat()   - 
Method in class sbrt.shell.io. AbstractMapInputFile 
 Returns the map format used to parse this file.
   getMapFormat()   - 
Method in class sbrt.shell.io. AbstractMapOutputFile 
 Returns the format used to write lines to this
 file.
   getMapFormat()   - 
Method in class sbrt.shell.io.fba. FluxCapInputFile 
 Returns the map format used to parse this file.
   getMapFormat()   - 
Method in interface sbrt.shell.io. MapTextFile 
 Returns the map format used by this file.
   getMatrix(IrrevRxnFluxome)   - 
Method in interface sbrt.kernel.fba.comp. ExtremeCurrentComputer 
 Returns the extreme current matrix for the provided fluxome.
   getMatrix(IrrevRxnFluxome, MutableProgressMonitor)   - 
Method in interface sbrt.kernel.fba.comp. ExtremeCurrentComputer 
 Returns the extreme current matrix for the provided fluxome.
   getMatrix(IrrevRxnFluxome)   - 
Method in class sbrt.kernel.fba.comp. SlpExCurrComputerV2 
 Returns the extreme current matrix for the provided fluxome.
   getMatrix(IrrevRxnFluxome, MutableProgressMonitor)   - 
Method in class sbrt.kernel.fba.comp. SlpExCurrComputerV2 
 Returns the extreme current matrix for the provided fluxome.
   getMatrix(ComiFile<String, Double>)   - 
Static method in class sbrt.shell.io. ComiFile 
 Reads the provided column-oriented map input file and returns its
 contents as a sparse matrix.
   getMax(MathExpr<?>)   - 
Method in class sbrt.kernel.fba.comp. FbaOptComputer 
 Returns the results of computing the maximum value of the 
 provided objective function.
   getMaxima(Collection<E>)   - 
Method in class sbrt.kernel.fba.comp. FbaOptComputer 
 Computes the maximum value of each provided objective function.
   getMaximum(String)   - 
Method in class sbrt.kernel.fba.comp. AbstractFluxIntervalComputer 
 Computes the maximum flux of the reaction with the provided name.
   getMaximum(MathExpr<String>)   - 
Method in class sbrt.kernel.fba.comp. AbstractFluxIntervalComputer 
 Computes the maximum value of the provided objective function.
   getMaximum(MathExpr<?>)   - 
Method in class sbrt.kernel.fba.comp. FbaOptComputer 
 Computes the maximum value of the provided objective function.
   getMaximumFluxes(Collection<String>, MutableProgressMonitor)   - 
Method in class sbrt.kernel.fba.comp. SimpleFluxIntervalComputer 
 Computes and returns the maximum fluxes of the reactions with the
 provided names.
   getMaxObstructedMoves(ManagerInput<String, String>, PrintWriter)   - 
Static method in class sbrt.shell.mng.geom. CdHarManager 
 Parses and returns the value for   CdHarManager.MAX_OBSTRUCTED_MOVES   in the
 provided manager input.
   getMaxWastedMoves()   - 
Method in class sbrt.kernel.fba.proc. CdHarFluxVectorProcess 
 Returns the allowed number of wasted moves.
   getMaxWithReverseConstrained(String)   - 
Method in class sbrt.kernel.fba.comp. AbstractFluxIntervalComputer 
 Computes the maximum flux of the reaction with the provided name while
 constraining the fluxes of its reverse reactions to their minimum.
   getMean()   - 
Method in class sbrt.kernel.math.stat. NormalDist 
 Returns the mean, or average, of this distribution.
   getMessage()   - 
Method in exception sbrt.shell. ApplicationException 
 Returns the error message of this process
 exception.
   getMin(MathExpr<?>)   - 
Method in class sbrt.kernel.fba.comp. FbaOptComputer 
 Returns the results of computing the minimum value of the 
 provided objective function.
   getMinChordLength()   - 
Method in class sbrt.kernel.fba.comp. CdHarFluxVectorComputer 
 Returns the minimum length a computed chord must have to be considered
 successful.
   getMinChordLength()   - 
Method in class sbrt.kernel.math.geom.comp. CdHarComputer 
 Returns the minimum length a computed chord must have to be considered
 successful.
   getMinChordLength(ManagerInput<String, String>, PrintWriter)   - 
Static method in class sbrt.shell.mng.geom. CdHarManager 
 Parses and returns the value for   CdHarManager.MIN_CHORD_LENGTH   in the
 provided manager input.
   getMinima(Collection<E>)   - 
Method in class sbrt.kernel.fba.comp. FbaOptComputer 
 Computes the minimum value of each provided objective function.
   getMinimum(String)   - 
Method in class sbrt.kernel.fba.comp. AbstractFluxIntervalComputer 
 Computes the minimum flux of the reaction with the provided name.
   getMinimum(MathExpr<String>)   - 
Method in class sbrt.kernel.fba.comp. AbstractFluxIntervalComputer 
 Computes the minimum value of the provided objective function.
   getMinimum(MathExpr<?>)   - 
Method in class sbrt.kernel.fba.comp. FbaOptComputer 
 Computes the minimum value of the provided objective function.
   getMulitComoFiles(ManagerInput<String, String>, PrintWriter, Iterator<String>, Fluxome)   - 
Static method in class sbrt.shell.mng.fba. SvCdHarFluxVectorManager 
 Returns a source of output files.
   getMultiColOrOutputFiles(ManagerInput<String, String>, PrintWriter, Iterator<String>, String, Fluxome)   - 
Static method in class sbrt.shell.mng.fba. FbaOptManager 
 Please consult the source code for a complete description of this
 method's actions.
   getMultiFileOutput(ManagerInput<String, String>, PrintWriter, Iterator<String>, String, Fluxome)   - 
Static method in class sbrt.shell.mng.fba. FbaOptManager 
 Please consult the source code for a complete description of this
 method's actions.
   getMultiFluxVectorOutputFile(ManagerInput<String, String>, PrintWriter, Fluxome)   - 
Static method in class sbrt.shell.mng.fba. CdHarFluxVectorManager 
 Returns an empty output file that is suitable for storing multiple flux
 vectors.
   getMultiFluxVectorOutputFile(ManagerInput<String, String>, PrintWriter, Fluxome)   - 
Static method in class sbrt.shell.mng.fba. FbaManager 
 Returns an empty output file that is suitable for storing multiple
 flux vectors.
   getMultiFluxVectorOutputFile(String, FileFormat, Set<String>, Fluxome)   - 
Static method in class sbrt.shell.mng.fba. FbaManager 
 Returns an empty output file that is suitable for storing multiple
 flux vectors.
   getMultiResultsOutputFile(ManagerInput<String, String>, PrintWriter, Fluxome)   - 
Static method in class sbrt.shell.mng.fba. FbaOptManager 
 Returns an empty output file that is suitable for storing multiple FBA
 optimization results.
   getMultiResultsOutputFile(String, FileFormat, Set<String>, Fluxome)   - 
Static method in class sbrt.shell.mng.fba. FbaOptManager 
 Returns an empty output file that is suitable for storing multiple FBA
 optimization results.
   getMultiResultsOutputFile(ManagerInput<String, String>, PrintWriter, Fluxome)   - 
Static method in class sbrt.shell.mng.fba. SimpleFluxIntervalManager 
 Returns an empty output file that is suitable for storing multiple sets
 of flux interval results.
   getMultiResultsOutputFile(String, Set<String>, Fluxome)   - 
Static method in class sbrt.shell.mng.fba. SimpleFluxIntervalManager 
 Returns an empty output file that is suitable for storing multiple sets
 of flux interval results.
   getMultiRowOrOutputFiles(ManagerInput<String, String>, PrintWriter, Iterator<String>, Fluxome)   - 
Static method in class sbrt.shell.mng.fba. FbaOptManager 
 Please consult the source code for a complete description of this
 method's actions.
   getMultiSerOutputFiles(ManagerInput<String, String>, PrintWriter, Iterator<String>, String, Fluxome)   - 
Static method in class sbrt.shell.mng.fba. FbaOptManager 
 Please consult the source code for a complete description of this
 method's actions.
   getN()   - 
Method in class sbrt.kernel.math.stat.comp. TauResult 
 Returns the sample size.
   getN1()   - 
Method in class sbrt.kernel.math.stat.comp. MannWhitneyResults 
 Returns the size of the larger sample.
   getN1(int, int)   - 
Method in class sbrt.kernel.math.stat.comp. MannWhitneyU 
 Returns the largest provided sample size.
   getN2()   - 
Method in class sbrt.kernel.math.stat.comp. MannWhitneyResults 
 Returns the size of the smaller sample.
   getN2(int, int)   - 
Method in class sbrt.kernel.math.stat.comp. MannWhitneyU 
 Returns the smallest provided sample size.
   getName()   - 
Method in class sbrt.kernel.io. AbstractResultsManager 
 Returns an empty string.
   getName()   - 
Method in interface sbrt.kernel.io. ResultsManager 
 Returns the name of this results manager.
   getName()   - 
Method in class sbrt.shell.io. AbstractMapOutputFile 
 Returns the canonical name of this file.
   getName()   - 
Method in class sbrt.shell.io.fba. ColumnOrFbaOptOutputFile 
 Returns the canonical name of this file.
   getName()   - 
Method in class sbrt.shell.io.fba. RowOrFbaOptOutputFile 
 Returns the canonical name of this file.
   getName()   - 
Method in class sbrt.shell.io.fba. SerFbaOptOutputFile 
 Returns the canonical name of this file.
   getName()   - 
Method in class sbrt.shell.io. FileFormat 
 Returns the name of this file format.
   getName()   - 
Method in class sbrt.shell.io. MultiOutputFiles 
 Returns the name of the current file.
   getName()   - 
Method in class sbrt.shell.io. ProcessInputFile 
 Returns the canonical name of this file.
   getName()   - 
Method in class sbrt.shell.io. SerializedOutputFile 
 Returns the canonical name of this file.
   getName()   - 
Method in class sbrt.shell.io. TextOutputFile 
 Returns the canonical name of this file.
   getName()   - 
Method in interface sbrt.shell.mng. ManagerInput 
 Returns the name of this manager input.
   getName()   - 
Method in class sbrt.shell.mng. MapManagerInput 
 Returns the name of this manager input.
   getNeighbors(Object)   - 
Method in class sbrt.kernel.math.graph_theory. DirectedGraph 
 Returns the neighboring nodes of the provided node.
   getNeverActive(Map<String, Interval>, double)   - 
Method in class sbrt.kernel.fba.analysis. FluxIntervalAnalyzer 
 Returns the never-active fluxes in the provided map.
   getNeverActive()   - 
Method in class sbrt.kernel.fba.proc. FluxPlasticityProcess.Results 
 Returns the set of fluxes that are never active.
   getNodes()   - 
Method in class sbrt.kernel.math.graph_theory. DirectedGraph 
 Returns the set of all nodes contained in this graph.
   getNumberFormat()   - 
Method in class sbrt.shell.text.math. CoeffFormatV1 
 Returns the format used for numerical values.
   getNumberFormat()   - 
Method in class sbrt.shell.text.math. CoeffFormatV2 
 Returns the format used for numerical values.
   getObjectiveFunction()   - 
Method in class sbrt.kernel.fba.comp. RxnDeletionComputer 
 Returns the current objective function.
   getObjectiveFunction()   - 
Method in class sbrt.kernel.fba.proc. CatalystDeletionProcess 
 Returns the current objective function.
   getObjectiveFunction()   - 
Method in class sbrt.kernel.fba.proc. CvCatalystDeletionProcess 
 Returns the current objective function.
   getObjectiveFunction()   - 
Method in class sbrt.kernel.fba.proc. CvFbaOptimizationExperiment 
 Returns the current objective function.
   getObjectiveFunction()   - 
Method in class sbrt.kernel.fba.proc. CvRxnDeletionProcess 
 Returns the current objective function.
   getObjectiveFunction()   - 
Method in class sbrt.kernel.fba.proc. FbaOptimizationProcess 
 Returns the current objective function.
   getObjectiveFunction()   - 
Method in class sbrt.kernel.fba.proc. RxnDeletionProcess 
 Returns the current objective function.
   getObjectiveFunction()   - 
Method in class sbrt.kernel.fba.proc. StoichVariationProcess 
 Returns the current objective function.
   getObjectiveFunction(ManagerInput<String, String>, PrintWriter, Fluxome)   - 
Static method in class sbrt.shell.mng.fba. FbaOptManager 
 Parses and returns the value for   FbaOptManager.OBJECTIVE_FUNCTION   in the
 provided manager input.
   getObjectiveValue()   - 
Method in class sbrt.ext.cplex. CplexSolver 
 Returns the most recently computed objective value.
   getObjectiveValue()   - 
Method in class sbrt.ext.glpk. GlpkSolver 
 Returns the most recently computed objective value.
   getObjectiveValue()   - 
Method in class sbrt.kernel.fba.comp. FbaOptComputer.Results 
 Returns the computed objective value.
   getObjectiveValue()   - 
Method in interface sbrt.kernel.math.optimization. ProgramSolver 
 Returns the most recently computed objective value.
   getObjFunction()   - 
Method in exception sbrt.kernel.fba.comp. FbaOptException 
 Returns the objective function whose optimal value could not be found.
   getObjValueHeader()   - 
Method in interface sbrt.shell.text.fba. FbaOptFileLineFormat 
 Returns the header used to indicate the value of the
 objective function.
   getObjValueHeader()   - 
Method in class sbrt.shell.text.fba. FbaOptFileLineFormatV1 
 Returns the header used to indicate the value of the
 objective function.
   getObjValueHeader()   - 
Method in interface sbrt.shell.text.fba. FbaOptHeaderFormatter 
 Returns the header used to indicate the value of the
 objective function.
   getObjValueHeader()   - 
Method in class sbrt.shell.text.fba. FbaOptHeaderFormatterV1 
 Returns the header used to indicate the value of the objective function.
   getObjValueHeader()   - 
Method in interface sbrt.shell.text.fba. FbaOptHeaderParser 
 Returns the header used to indicate the value of the
 objective function.
   getObjValueHeader()   - 
Method in class sbrt.shell.text.fba. FbaOptHeaderParserV1 
 Returns the header used to indicate the value of the objective function.
   getObsructedMovesFile(ManagerInput<String, String>, PrintWriter)   - 
Static method in class sbrt.shell.mng.fba. SvCdHarFluxVectorManager 
 Returns an output file used to store the number of obstructed moves per
 flux space.
   getObstructedMovesFile(String)   - 
Static method in class sbrt.shell.mng.fba. SvCdHarFluxVectorManager 
 Returns an output file used to store the number of obstructed moves per
 flux space.
   getOffender()   - 
Method in exception sbrt.kernel.fba.comp. FluxRangeException 
 Returns the object whose range has exceeded the defined cutoff.
   getOnFluxes(ManagerInput<String, String>, PrintWriter)   - 
Static method in class sbrt.shell.mng.fba. RandomConstraintsManager 
 Parses and returns the value for   RandomConstraintsManager.NUMBER_OF_ON_FLUXES   in the 
 provided manager input.
   getOptimal(String, OptimizationSense)   - 
Method in class sbrt.kernel.fba.comp. FbaOptComputer 
 Returns the results of computing the optimal flux of the reaction 
 with the provided name.
   getOptimal(MathExpr<?>, OptimizationSense)   - 
Method in class sbrt.kernel.fba.comp. FbaOptComputer 
 Returns the results of computing the optimal value of the 
 provided objective function.
   getOptimizationSense()   - 
Method in class sbrt.kernel.fba.comp. RxnDeletionComputer 
 Returns the current optimization sense.
   getOptimizationSense()   - 
Method in class sbrt.kernel.fba.proc. CatalystDeletionProcess 
 Returns the current optimization sense.
   getOptimizationSense()   - 
Method in class sbrt.kernel.fba.proc. CvCatalystDeletionProcess 
 Returns the current optimization sense.
   getOptimizationSense()   - 
Method in class sbrt.kernel.fba.proc. CvFbaOptimizationExperiment 
 Returns the current optimization sense.
   getOptimizationSense()   - 
Method in class sbrt.kernel.fba.proc. CvObjectiveFunctionProcess 
 Returns the current optimization sense.
   getOptimizationSense()   - 
Method in class sbrt.kernel.fba.proc. CvRxnDeletionProcess 
 Returns the current optimization sense.
   getOptimizationSense()   - 
Method in class sbrt.kernel.fba.proc. FbaOptimizationProcess 
 Returns the current optimization sense.
   getOptimizationSense()   - 
Method in class sbrt.kernel.fba.proc. ObjectiveFunctionAnalysis 
 Returns the current optimization sense.
   getOptimizationSense()   - 
Method in class sbrt.kernel.fba.proc. RxnDeletionProcess 
 Returns the current optimization sense.
   getOptimizationSense()   - 
Method in class sbrt.kernel.fba.proc. StoichVariationProcess 
 Returns the current optimization sense.
   getOptimizationSense(ManagerInput<String, String>)   - 
Static method in class sbrt.shell.mng.fba. FbaOptManager 
 Parses and returns the value for   FbaOptManager.OPTIMIZATION_SENSE   in the
 provided manager input.
   getOptionalKeys()   - 
Method in class sbrt.shell.mng.algebra. LinearCombConverterManager 
 Returns the set of keywords understood, but not required, by this process
 manager.
   getOptionalKeys()   - 
Method in class sbrt.shell.mng.algebra. LinearSolverManager 
 Returns the set of keywords understood, but not required, by this process
 manager.
   getOptionalKeys()   - 
Method in class sbrt.shell.mng.algebra. MatrixConverterManager 
 Returns the set of keywords understood, but not required, by this process
 manager.
   getOptionalKeys()   - 
Method in class sbrt.shell.mng.algebra. MultipleVectorsConverterManager 
 Returns the set of keywords understood, but not required, by this process
 manager.
   getOptionalKeys()   - 
Method in class sbrt.shell.mng.algebra. SingleVectorConverterManager 
 Returns the set of keywords understood, but not required, by this process
 manager.
   getOptionalKeys()   - 
Method in class sbrt.shell.mng.comb. SingleElementUnionManager 
 Returns the set of keywords understood, but not required, by this process
 manager.
   getOptionalKeys()   - 
Method in class sbrt.shell.mng.comb. StrictSingleElementUnionManager 
 Returns the set of keywords understood, but not required, by this process
 manager.
   getOptionalKeys()   - 
Method in class sbrt.shell.mng.fba. BiggSbmlReaderManager 
 Returns the set of keywords understood, but not required, by this process
 manager.
   getOptionalKeys()   - 
Method in class sbrt.shell.mng.fba. BiggSbmlTranslaterManager 
 Returns the set of keywords understood, but not required, by this process
 manager.
   getOptionalKeys()   - 
Method in class sbrt.shell.mng.fba. CatalystDeletionManager 
 Returns the set of keywords the provided
  ManagerInput  object does not necessarily
 have to contain.
   getOptionalKeys()   - 
Method in class sbrt.shell.mng.fba. CdHarFluxVectorManager 
 Returns the set of keywords the provided  ManagerInput  object does
 not necessarily have to contain.
   getOptionalKeys()   - 
Method in class sbrt.shell.mng.fba. CvCatalystDeletionManager 
 Returns the set of keywords the provided  ManagerInput  object does
 not necessarily have to contain.
   getOptionalKeys()   - 
Method in class sbrt.shell.mng.fba. CvFbaOptManager 
 Returns the set of keywords the provided
  ManagerInput  object does not necessarily
 have to contain.
   getOptionalKeys()   - 
Method in class sbrt.shell.mng.fba. CvMsFluxIntervalManager 
 Returns the set of keywords the provided
  ManagerInput  object does not necessarily
 have to contain.
   getOptionalKeys()   - 
Method in class sbrt.shell.mng.fba. CvObjectiveFunctionManager 
 Returns the set of keywords the provided
  ManagerInput  object does not necessarily
 have to contain.
   getOptionalKeys()   - 
Method in class sbrt.shell.mng.fba. CvRevRxnFluxIntervalManager 
 Returns the set of keywords the provided  ManagerInput  object does
 not necessarily have to contain.
   getOptionalKeys()   - 
Method in class sbrt.shell.mng.fba. CvRxnDeletionManager 
 Returns the set of keywords the provided
  ManagerInput  object does not necessarily
 have to contain.
   getOptionalKeys()   - 
Method in class sbrt.shell.mng.fba. CvSimpleFluxIntervalManager 
 Returns the set of keywords the provided
  ManagerInput  object does not necessarily
 have to contain.
   getOptionalKeys()   - 
Method in class sbrt.shell.mng.fba. ExtremeCurrentManager 
 Returns the set of keywords the provided
  ManagerInput  object does not necessarily
 have to contain.
   getOptionalKeys()   - 
Method in class sbrt.shell.mng.fba. FbaOptManager 
 Returns the set of keywords the provided  ManagerInput  object does
 not necessarily have to contain.
   getOptionalKeys()   - 
Method in class sbrt.shell.mng.fba. FbaSolverManager 
 Returns the set of keywords the provided
  ManagerInput  object does not necessarily
 have to contain.
   getOptionalKeys()   - 
Method in class sbrt.shell.mng.fba. FluxActivityManager 
 Returns the set of keywords the provided  ManagerInput  object does
 not necessarily have to contain.
   getOptionalKeys()   - 
Method in class sbrt.shell.mng.fba. FluxCapManager 
 Returns the set of keywords the provided
  ManagerInput  object does not necessarily
 have to contain.
   getOptionalKeys()   - 
Method in class sbrt.shell.mng.fba. FluxPlasticityManager 
 Returns the set of keywords the provided  ManagerInput  object does
 not necessarily have to contain.
   getOptionalKeys()   - 
Method in class sbrt.shell.mng.fba. InitialPointManager 
 Returns the set of keywords the provided  ManagerInput  object does
 not necessarily have to contain.
   getOptionalKeys()   - 
Method in class sbrt.shell.mng.fba. MetatoolFileWriterManager 
 Returns the set of keywords understood, but not required, by this process
 manager.
   getOptionalKeys()   - 
Method in class sbrt.shell.mng.fba. MmaNotebookManager 
 Returns the set of keywords the provided  ManagerInput  object does
 not necessarily have to contain.
   getOptionalKeys()   - 
Method in class sbrt.shell.mng.fba. MsFluxIntervalManager 
 Returns the set of keywords the provided  ManagerInput  object does
 not necessarily have to contain.
   getOptionalKeys()   - 
Method in class sbrt.shell.mng.fba. MsFluxomeReducerManager 
 Returns the set of keywords the provided
  ManagerInput  object does not necessarily
 have to contain.
   getOptionalKeys()   - 
Method in class sbrt.shell.mng.fba. NetworkInfoManager 
 Returns the set of keywords the provided  ManagerInput  object does
 not necessarily have to contain.
   getOptionalKeys()   - 
Method in class sbrt.shell.mng.fba. ObjectiveFunctionManager 
 Returns the set of keywords the provided
  ManagerInput  object does not necessarily
 have to contain.
   getOptionalKeys()   - 
Method in class sbrt.shell.mng.fba. PalssonSbmlReaderManager 
 Returns the set of keywords understood, but not required, by this process
 manager.
   getOptionalKeys()   - 
Method in class sbrt.shell.mng.fba. PalssonSbmlTranslaterManager 
 Returns the set of keywords understood, but not required, by this process
 manager.
   getOptionalKeys()   - 
Method in class sbrt.shell.mng.fba. RandomConstraintsManager 
 Returns the set of keywords the provided
  ManagerInput  object does not necessarily
 have to contain.
   getOptionalKeys()   - 
Method in class sbrt.shell.mng.fba. RandomEnvManager 
 Returns the set of keywords the provided  ManagerInput  object does
 not necessarily have to contain.
   getOptionalKeys()   - 
Method in class sbrt.shell.mng.fba. RandomObjectiveManager 
 Returns the set of keywords the provided
  ManagerInput  object does not necessarily
 have to contain.
   getOptionalKeys()   - 
Method in class sbrt.shell.mng.fba. ReverseRxnFluxIntervalManager 
 Returns the set of keywords the provided  ManagerInput  object does
 not necessarily have to contain.
   getOptionalKeys()   - 
Method in class sbrt.shell.mng.fba. RxnDeletionManager 
 Returns the set of keywords the provided
  ManagerInput  object does not necessarily
 have to contain.
   getOptionalKeys()   - 
Method in class sbrt.shell.mng.fba. RxnRemovalManager 
 Returns the set of keywords the provided  ManagerInput  object does
 not necessarily have to contain.
   getOptionalKeys()   - 
Method in class sbrt.shell.mng.fba. SbmlWriterManager 
 Returns the set of keywords understood, but not required, by this process
 manager.
   getOptionalKeys()   - 
Method in class sbrt.shell.mng.fba. SimpleFluxIntervalManager 
 Returns the set of keywords the provided  ManagerInput  object does
 not necessarily have to contain.
   getOptionalKeys()   - 
Method in class sbrt.shell.mng.fba. SimpleRxnFileTransManager 
 Returns the set of keywords understood, but not required, by this process
 manager.
   getOptionalKeys()   - 
Method in class sbrt.shell.mng.fba. SlpCycleIdManager 
 Returns the set of keywords the provided  ManagerInput  object does
 not necessarily have to contain.
   getOptionalKeys()   - 
Method in class sbrt.shell.mng.fba. StoichVariationManager 
 Returns the set of keywords the provided  ManagerInput  object does
 not necessarily have to contain.
   getOptionalKeys()   - 
Method in class sbrt.shell.mng.fba. SvCdHarFluxVectorManager 
 Returns the set of keywords the provided  ManagerInput  object does
 not necessarily have to contain.
   getOptionalKeys()   - 
Method in class sbrt.shell.mng.fba. SvInitialPointManager 
 Returns the set of keywords the provided  ManagerInput  object does
 not necessarily have to contain.
   getOptionalKeys()   - 
Method in class sbrt.shell.mng.fba. WwFluxomeReducerManager 
 Returns the set of keywords the provided
  ManagerInput  object does not necessarily
 have to contain.
   getOptionalKeys()   - 
Method in class sbrt.shell.mng.geom. CdHarManager 
 Returns the set of keywords understood, but not required, by this process
 manager.
   getOptionalKeys()   - 
Method in class sbrt.shell.mng.graph_theory. CycleIdManager 
 Returns the set of keywords the provided
  ManagerInput  object does not necessarily
 have to contain.
   getOptionalKeys()   - 
Method in class sbrt.shell.mng.graph_theory. PathIdManager 
 Returns the set of keywords the provided
  ManagerInput  object does not necessarily
 have to contain.
   getOptionalKeys()   - 
Method in class sbrt.shell.mng.graph_theory. UniqueCycleIdManager 
 Returns the set of keywords the provided
  ManagerInput  object does not necessarily
 have to contain.
   getOptionalKeys()   - 
Method in interface sbrt.shell.mng. ProcessManager 
 Returns the set of keys the provided
  ManagerInput  object does not necessarily
 have to contain.
   getOptionalKeys()   - 
Method in class sbrt.shell.mng.stat. CorrelationManager 
 Returns the set of keywords understood, but not required, by this process
 manager.
   getOptionalKeys()   - 
Method in class sbrt.shell.mng.stat. KendallTauManager 
 Returns the set of keywords understood, but not required, by this 
 process manager.
   getOptionalKeys()   - 
Method in class sbrt.shell.mng.stat. MannWhitneyUManager 
 Returns the set of keywords understood, but not required, by this
 process manager.
   getOptionalKeys()   - 
Method in class sbrt.shell.mng.utilities. IntervalComparisonManager 
 Returns the set of keywords understood, but not required, by this process
 manager.
   getOptionalKeys()   - 
Method in class sbrt.shell.mng.utilities. VariableParticipationManager 
 Returns the set of keywords the provided
  ManagerInput  object does not necessarily
 have to contain.
   getOptionalKeys()   - 
Method in class sbrt.shell.mng.utilities. VectorComparisonManager 
 Returns the set of keywords understood, but not required, by this process
 manager.
   getOptionalValue(Object)   - 
Method in class sbrt.shell.io. ProcessInputFile 
 Returns the value corresponding to the provided key.
   getOptionalValue(Object)   - 
Method in interface sbrt.shell.mng. ManagerInput 
 Returns the value corresponding to the provided key.
   getOptionalValue(Object)   - 
Method in class sbrt.shell.mng. MapManagerInput 
 Returns the value corresponding to the provided key.
   getOriginalName()   - 
Method in class sbrt.shell.io. AbstractMapInputFile 
 Returns the name of this file.
   getOriginalName()   - 
Method in class sbrt.shell.io. AbstractMapOutputFile 
 Returns the name of this file.
   getOriginalName()   - 
Method in class sbrt.shell.io.fba. ColumnOrFbaOptOutputFile 
 Returns the name of this file.
   getOriginalName()   - 
Method in class sbrt.shell.io.fba. FluxCapInputFile 
 Returns the name of this file.
   getOriginalName()   - 
Method in class sbrt.shell.io.fba. FluxIntervalInputFile 
 Returns the name of this file.
   getOriginalName()   - 
Method in class sbrt.shell.io.fba. RowOrFbaOptOutputFile 
 Returns the name of this file.
   getOriginalName()   - 
Method in class sbrt.shell.io.fba. SerFbaOptOutputFile 
 Returns the name of this file.
   getOriginalName()   - 
Method in interface sbrt.shell.io. SbrtFile 
 Returns the original name given to this file.
   getOriginalName()   - 
Method in class sbrt.shell.io. SerializedInputFile 
 Returns the original name given to this file object.
   getOriginalName()   - 
Method in class sbrt.shell.io. SerializedOutputFile 
 Returns the name of this file.
   getOriginalName()   - 
Method in class sbrt.shell.io. TextInputFile 
 Returns the name of this file.
   getOriginalName()   - 
Method in class sbrt.shell.io. TextOutputFile 
 Returns the name of this file.
   getOutputFile(ManagerInput<String, String>, PrintWriter)   - 
Static method in class sbrt.shell.mng.comb. SingleElementUnionManager 
 Returns an output file with the name provided in the manager input.
   getOutputFile(String, PrintWriter)   - 
Static method in class sbrt.shell.mng.comb. SingleElementUnionManager 
 Returns an output file with the provided name.
   getOutputFile(String)   - 
Static method in class sbrt.shell.mng.comb. SingleElementUnionManager 
 Returns an output file with the provided name.
   getOutputFile(ManagerInput<String, String>, PrintWriter, Fluxome, Set<String>)   - 
Static method in class sbrt.shell.mng.fba. ExtremeCurrentManager 
 Returns an output file to which extreme currents can be written.
   getOutputFile(String, PrintWriter, Fluxome, Set<String>)   - 
Static method in class sbrt.shell.mng.fba. ExtremeCurrentManager 
 Returns an output file to which extreme currents can be written.
   getOutputFile(ManagerInput<String, String>, PrintWriter, IrrevRxnFluxome)   - 
Static method in class sbrt.shell.mng.fba. RandomConstraintsManager 
 Returns an empty output file that is suitable for storing multiple
 sets of flux constraints.
   getOutputFile(String, FileFormat, Set<String>, IrrevRxnFluxome)   - 
Static method in class sbrt.shell.mng.fba. RandomConstraintsManager 
 Returns an empty output file that is suitable for storing multiple
 sets of flux constraints.
   getOutputFile(String, Set<String>, IrrevRxnFluxome)   - 
Static method in class sbrt.shell.mng.fba. RandomConstraintsManager 
 Returns an empty output file that is suitable for storing multiple
 sets of flux constraints.
   getOutputFile(ManagerInput<String, String>, PrintWriter, IrrevRxnFluxome)   - 
Static method in class sbrt.shell.mng.fba. RandomEnvManager 
 Returns an empty output file that is suitable for storing multiple environments.
   getOutputFile(String, FileFormat, PrintWriter, IrrevRxnFluxome)   - 
Static method in class sbrt.shell.mng.fba. RandomObjectiveManager 
 Returns an empty output file that is suitable for storing randomly 
 generated objective functions.
   getOutputFile(ManagerInput<String, String>, PrintWriter, IrrevRxnFluxome)   - 
Static method in class sbrt.shell.mng.fba. RandomObjectiveManager 
 Returns an empty output file that is suitable for storing randomly
 generated objective functions.
   getOutputFile(String)   - 
Static method in class sbrt.shell.mng.fba. WwFluxomeReducerManager 
 Returns a file to which the chemical reactions of a fluxome can be 
 written.
   getOutputFile(String, PrintWriter)   - 
Static method in class sbrt.shell.mng.fba. WwFluxomeReducerManager 
 Returns a file to which the chemical reactions of a fluxome can be 
 written.
   getOutputFile(ManagerInput<String, String>, PrintWriter, Set<String>)   - 
Static method in class sbrt.shell.mng.geom. CdHarManager 
 Returns an output file with the name provided in the manager input.
   getOutputFile(String, PrintWriter, Set<String>)   - 
Static method in class sbrt.shell.mng.geom. CdHarManager 
 Returns an output file with the provided name.
   getOutputFile(String, Set<String>)   - 
Static method in class sbrt.shell.mng.geom. CdHarManager 
 Returns an output file with the provided name.
   getOutputFile(ManagerInput<String, String>, PrintWriter)   - 
Static method in class sbrt.shell.mng.graph_theory. CycleIdManager 
 Creates a returns an output file if one is specified in the provided
 input.
   getOutputFile(ManagerInput<String, String>)   - 
Static method in class sbrt.shell.mng.graph_theory. CycleIdManager 
 Creates a returns an output file if one is specified in the provided
 input.
   getOutputIrrevRxnFormat()   - 
Method in class sbrt.shell.proc.fba. SimpleRxnFileTranslator 
 Returns the formatter used for irreversible reactions in the
 output file.
   getP()   - 
Method in class sbrt.ext.R.stat. RCorrelator.Result 
 Returns the corresponding P value.
   getPaths()   - 
Method in class sbrt.kernel.math.graph_theory.comp. PathFinder 
 Returns a list of all paths.
   getPaths(Collection<? extends N>, Collection<? extends N>)   - 
Method in class sbrt.kernel.math.graph_theory.comp. PathFinder 
 Returns a list of all paths between the provided source and sink nodes.
   getPaths(T, S)   - 
Method in class sbrt.kernel.math.graph_theory.comp. PathFinder 
 Returns a list of all paths between the provided source and sink nodes.
   getPIntercept()   - 
Method in class sbrt.kernel.fba.comp. InitialPointComputer 
 Returns the value of each positioning variable at the edges
 of the convex polytope.
   getPIntercept(ManagerInput<String, String>, PrintWriter)   - 
Static method in class sbrt.shell.mng.fba. InitialPointManager 
 Returns the parsed value for   InitialPointManager.P_MAX   in the provided input.
   getPipeSetFormat()   - 
Method in class sbrt.shell.text.fba. FbaOptHeaderFormatterV1 
 Returns the format used for sets of strings.
   getPipeSetFormat()   - 
Method in class sbrt.shell.text.fba. FbaOptHeaderParserV1 
 Returns the format used for sets of strings.
   getPlane()   - 
Method in class sbrt.kernel.math.geom. ParallelHyperplanes 
 Returns the hyperplane.
   getPlanes(String, PrintWriter)   - 
Static method in class sbrt.shell.mng.geom. CdHarManager 
 Returns the list of sets contained in the file with the provided name.
   getPlanes(String)   - 
Static method in class sbrt.shell.mng.geom. CdHarManager 
 Returns the list of sets contained in the file with the provided name.
   getPlanes(ManagerInput<String, String>, PrintWriter)   - 
Static method in class sbrt.shell.mng.geom. CdHarManager 
 Returns the list of sets contained in the file with the name contained in
 the provided input.
   getPoint()   - 
Method in class sbrt.kernel.math.geom.comp. CdHarComputer.Results 
 Returns the random interior point that was computed.
   getPoint()   - 
Method in exception sbrt.kernel.math. InvalidPointException 
 Returns the invalid point.
   getPointsPerPoint()   - 
Method in class sbrt.kernel.fba.proc. CdHarFluxVectorProcess 
 Returns the number of points generated per points returned.
   getPointsPerPoint(ManagerInput<String, String>, PrintWriter)   - 
Static method in class sbrt.shell.mng.geom. CdHarManager 
 Parses and returns the value for   CdHarManager.POINTS_PER_POINT   in the
 provided manager input.
   getPolytope()   - 
Method in class sbrt.kernel.math.geom.comp. CdHarComputer 
 Returns the convex polytope for which random points are generated.
   getPotActive()   - 
Method in class sbrt.kernel.fba.proc. FluxPlasticityProcess.Results 
 Returns the set of fluxes that are potentially active.
   getPotentiallyActive(Map<String, Interval>, double)   - 
Method in class sbrt.kernel.fba.analysis. FluxIntervalAnalyzer 
 Returns the potentially-active fluxes in the provided map.
   getPredecessors(Object)   - 
Method in class sbrt.kernel.math.graph_theory. DirectedGraph 
 Returns the predecessors of the provided node.
   getProcess()   - 
Method in class sbrt.shell.mng.algebra. LinearSolverManager 
 Returns the process controlled by this process manager.
   getProcess()   - 
Method in class sbrt.shell.mng.comb. SingleElementUnionManager 
 Returns the process controlled by this process manager.
   getProcess()   - 
Method in class sbrt.shell.mng.comb. StrictSingleElementUnionManager 
 Returns the process controlled by this process manager.
   getProcess()   - 
Method in class sbrt.shell.mng.fba. CatalystDeletionManager 
 Returns the process this process manager controls.
   getProcess()   - 
Method in class sbrt.shell.mng.fba. CdHarFluxVectorManager 
 Returns the process this process manager controls.
   getProcess()   - 
Method in class sbrt.shell.mng.fba. CvCatalystDeletionManager 
 Returns the process this process manager controls.
   getProcess()   - 
Method in class sbrt.shell.mng.fba. CvFbaOptManager 
 Returns the process this process manager controls.
   getProcess()   - 
Method in class sbrt.shell.mng.fba. CvMsFluxIntervalManager 
 Returns the process this process manager controls.
   getProcess()   - 
Method in class sbrt.shell.mng.fba. CvObjectiveFunctionManager 
 Returns the process this process manager controls.
   getProcess()   - 
Method in class sbrt.shell.mng.fba. CvRevRxnFluxIntervalManager 
 Returns the process this process manager controls.
   getProcess()   - 
Method in class sbrt.shell.mng.fba. CvRxnDeletionManager 
 Returns the process this process manager controls.
   getProcess()   - 
Method in class sbrt.shell.mng.fba. CvSimpleFluxIntervalManager 
 Returns the process this process manager controls.
   getProcess()   - 
Method in class sbrt.shell.mng.fba. ExtremeCurrentManager 
 Returns the process this process manager controls.
   getProcess()   - 
Method in class sbrt.shell.mng.fba. FbaOptManager 
 Returns the process this process manager controls.
   getProcess()   - 
Method in class sbrt.shell.mng.fba. FbaSolverManager 
 Returns the process this process manager controls.
   getProcess()   - 
Method in class sbrt.shell.mng.fba. FluxActivityManager 
 Returns the process this process manager controls.
   getProcess()   - 
Method in class sbrt.shell.mng.fba. FluxCapManager 
 Returns the process this process manager controls.
   getProcess()   - 
Method in class sbrt.shell.mng.fba. FluxPlasticityManager 
 Returns the process this process manager controls.
   getProcess()   - 
Method in class sbrt.shell.mng.fba. InitialPointManager 
 Returns the process this process manager controls.
   getProcess()   - 
Method in class sbrt.shell.mng.fba. MsFluxIntervalManager 
 Returns the process this process manager controls.
   getProcess()   - 
Method in class sbrt.shell.mng.fba. MsFluxomeReducerManager 
 Returns the process this process manager controls.
   getProcess()   - 
Method in class sbrt.shell.mng.fba. NetworkInfoManager 
 Returns the process this process manager controls.
   getProcess()   - 
Method in class sbrt.shell.mng.fba. ObjectiveFunctionManager 
 Returns the process this process manager controls.
   getProcess()   - 
Method in class sbrt.shell.mng.fba. RandomConstraintsManager 
 Returns the process this process manager controls.
   getProcess()   - 
Method in class sbrt.shell.mng.fba. RandomEnvManager 
 Returns the process this process manager controls.
   getProcess()   - 
Method in class sbrt.shell.mng.fba. RandomObjectiveManager 
 Returns the process this process manager controls.
   getProcess()   - 
Method in class sbrt.shell.mng.fba. ReverseRxnFluxIntervalManager 
 Returns the process this process manager controls.
   getProcess()   - 
Method in class sbrt.shell.mng.fba. RxnDeletionManager 
 Returns the process this process manager controls.
   getProcess()   - 
Method in class sbrt.shell.mng.fba. SimpleFluxIntervalManager 
 Returns the process this process manager controls.
   getProcess()   - 
Method in class sbrt.shell.mng.fba. SlpCycleIdManager 
 Returns the process this process manager controls.
   getProcess()   - 
Method in class sbrt.shell.mng.fba. StoichVariationManager 
 Returns the process this process manager controls.
   getProcess()   - 
Method in class sbrt.shell.mng.fba. SvCdHarFluxVectorManager 
 Returns the process this process manager controls.
   getProcess()   - 
Method in class sbrt.shell.mng.fba. SvInitialPointManager 
 Returns the process this process manager controls.
   getProcess()   - 
Method in class sbrt.shell.mng.fba. WwFluxomeReducerManager 
 Returns the process this process manager controls.
   getProcess()   - 
Method in class sbrt.shell.mng.geom. CdHarManager 
 Returns the process controlled by this process manager.
   getProcess()   - 
Method in class sbrt.shell.mng.graph_theory. CycleIdManager 
 Returns the process this process manager controls.
   getProcess()   - 
Method in class sbrt.shell.mng.graph_theory. PathIdManager 
 Returns the process this process manager controls.
   getProcess()   - 
Method in interface sbrt.shell.mng. KernelProcessManager 
 Returns the process this process manager controls.
   getProcess()   - 
Method in class sbrt.shell.mng.stat. CorrelationManager 
 Returns the process controlled by this process manager.
   getProcess()   - 
Method in class sbrt.shell.mng.stat. KendallTauManager 
 Returns the process controlled by this process manager.
   getProcess()   - 
Method in class sbrt.shell.mng.stat. MannWhitneyUManager 
 Returns the process controlled by this process manager.
   getProcess()   - 
Method in class sbrt.shell.mng.utilities. IntervalComparisonManager 
 Returns the process this process manager controls.
   getProcess()   - 
Method in class sbrt.shell.mng.utilities. VariableParticipationManager 
 Returns the process this process manager controls.
   getProcess()   - 
Method in class sbrt.shell.mng.utilities. VectorComparisonManager 
 Returns the process managed by this object.
   getProducts()   - 
Method in class sbrt.kernel.fba. AbstractRxn 
 Returns the products of this reaction.
   getProducts()   - 
Method in interface sbrt.kernel.fba. ChemRxn 
 Returns the products of this chemical reaction as a 
 linear combination of chemical species.
   getProducts()   - 
Method in class sbrt.kernel.fba. ExchangeRxn 
 Returns an empty linear combination.
   getProducts()   - 
Method in class sbrt.kernel.fba. SourceRxn 
 Returns a single-term linear combination.
   getProducts(Node)   - 
Method in class sbrt.shell.text.fba. BiggRxnNodeParser 
 Returns the products of the provided reaction node.
   getProducts(Node)   - 
Method in class sbrt.shell.text.fba. PalssonRxnNodeParser 
 Returns the products of the provided reaction node.
   getProgramSolverType()   - 
Method in class sbrt.kernel.fba.comp. FbaOptComputer 
 Returns the  Class  of the program solver used by this 
 optimization computer.
   getProgress()   - 
Method in interface sbrt.kernel.fba.comp. CycleComputer 
 Returns a progress monitor for this cycle computer.
   getProgress()   - 
Method in interface sbrt.kernel.fba.comp. ExtremeCurrentComputer 
 Returns a progress monitor for this extreme current computer.
   getProgress()   - 
Method in class sbrt.kernel.fba.comp. FastMsFluxRangeComputer 
 Returns a progress monitor for this flux interval computer.
   getProgress()   - 
Method in class sbrt.kernel.fba.comp. FluxCapComputer 
 Returns a progress monitor for this flux cap computer.
   getProgress()   - 
Method in interface sbrt.kernel.fba.comp. FluxIntervalComputer 
 Returns a progress monitor for this flux interval computer.
   getProgress()   - 
Method in interface sbrt.kernel.fba.comp. FluxomeReducer 
 Returns a progress monitor for this fluxome reducer.
   getProgress()   - 
Method in class sbrt.kernel.fba.comp. MsFluxIntervalComputer 
 Returns a progress monitor for this flux interval computer.
   getProgress()   - 
Method in class sbrt.kernel.fba.comp. MsFluxomeReducer 
 Returns a progress monitor for this fluxome reducer.
   getProgress()   - 
Method in class sbrt.kernel.fba.comp. ReverseRxnFluxIntervalComputer 
 Returns a progress monitor for this flux interval computer.
   getProgress()   - 
Method in class sbrt.kernel.fba.comp. SimpleFluxIntervalComputer 
 Returns a progress monitor for this flux interval computer.
   getProgress()   - 
Method in class sbrt.kernel.fba.comp. SlpCycleComputer 
 Returns a progress monitor for this steady state cycle computer.
   getProgress()   - 
Method in class sbrt.kernel.fba.comp. SlpExCurrComputerV2 
 Returns a progress monitor for this extreme current computer.
   getProgress()   - 
Method in class sbrt.kernel.fba.comp. WwFluxomeReducer 
 Returns a progress monitor for this fluxome reducer.
   getProgress()   - 
Method in class sbrt.kernel.fba.proc. CatalystDeletionProcess 
 Returns a progress monitor for this experiment.
   getProgress()   - 
Method in class sbrt.kernel.fba.proc. CdHarFluxVectorProcess 
 Returns a progress monitor for this experiment.
   getProgress()   - 
Method in class sbrt.kernel.fba.proc. CvCatalystDeletionProcess 
 Returns a progress monitor for this experiment.
   getProgress()   - 
Method in class sbrt.kernel.fba.proc. CvFbaOptimizationExperiment 
 Returns a progress monitor for this experiment.
   getProgress()   - 
Method in class sbrt.kernel.fba.proc. CvFluxIntervalProcess 
 Returns a progress monitor for this experiment.
   getProgress()   - 
Method in class sbrt.kernel.fba.proc. CvObjectiveFunctionProcess 
 Returns a progress monitor for this experiment.
   getProgress()   - 
Method in class sbrt.kernel.fba.proc. CvRxnDeletionProcess 
 Returns a progress monitor for this experiment.
   getProgress()   - 
Method in class sbrt.kernel.fba.proc. CycleIdProcess 
 Returns a progress monitor for this process.
   getProgress()   - 
Method in class sbrt.kernel.fba.proc. ExtremeCurrentProcess 
 Returns a progress monitor for this experiment.
   getProgress()   - 
Method in class sbrt.kernel.fba.proc. FluxActivityProcess 
 Returns a progress monitor for this experiment.
   getProgress()   - 
Method in class sbrt.kernel.fba.proc. FluxCapProcess 
 Returns a progress monitor for this experiment.
   getProgress()   - 
Method in class sbrt.kernel.fba.proc. FluxIntervalProcess 
 Returns a progress monitor for this experiment.
   getProgress()   - 
Method in class sbrt.kernel.fba.proc. FluxomeReducerProcess 
 Returns a progress monitor for this process.
   getProgress()   - 
Method in class sbrt.kernel.fba.proc. FluxPlasticityProcess 
 Returns a progress monitor for this experiment.
   getProgress()   - 
Method in class sbrt.kernel.fba.proc. ObjectiveFunctionAnalysis 
 Returns a progress monitor for this experiment.
   getProgress()   - 
Method in class sbrt.kernel.fba.proc. RandomConstraintsGeneratorV1 
 Returns a progress monitor for this process.
   getProgress()   - 
Method in class sbrt.kernel.fba.proc. RandomEnvGeneratorV1 
 Returns a progress monitor for this process.
   getProgress()   - 
Method in class sbrt.kernel.fba.proc. RandomObjectiveGenerator 
 Returns a progress monitor for this process.
   getProgress()   - 
Method in class sbrt.kernel.fba.proc. RxnDeletionProcess 
 Returns a progress monitor for this experiment.
   getProgress()   - 
Method in class sbrt.kernel.fba.proc. StoichVariationProcess 
 Returns a progress monitor for this process.
   getProgress()   - 
Method in class sbrt.kernel.fba.proc. SvCdHarFluxVectorProcess 
 Returns a progress monitor for this experiment.
   getProgress()   - 
Method in class sbrt.kernel.fba.proc. SvInitialPointProcess 
 Returns a progress monitor for this experiment.
   getProgress()   - 
Method in class sbrt.kernel.math.geom.proc. CdHarProcess 
 Returns a progress monitor for this process.
   getProgress()   - 
Method in class sbrt.kernel.math.graph_theory.proc. CycleIdProcess 
 Returns a progress monitor for this experiment.
   getProgress()   - 
Method in class sbrt.kernel.math.graph_theory.proc. PathIdProcess 
 Returns a progress monitor for this experiment.
   getProgress()   - 
Method in class sbrt.kernel.math.stat.proc. MultipleCorrelationAnalysis 
 Returns a progress monitor for this process.
   getProgress()   - 
Method in interface sbrt.kernel. MonitoredProcess 
 Returns a progress monitor for this process.
   getProgress()   - 
Method in class sbrt.kernel.util. DiscreteProgressMonitor 
 Returns an integer value indicating a progress.
   getProgress()   - 
Method in class sbrt.kernel.util. PercentProgressMonitor 
 Returns a double precision number indicating the completed percentage 
 of the process being monitored.
   getProgress()   - 
Method in interface sbrt.kernel.util. ProgressMonitor 
 Returns a number indicating the progress of the process being monitored.
   getProgress()   - 
Method in class sbrt.kernel.utilities.proc. IntervalComparison 
  
   getProgress()   - 
Method in class sbrt.kernel.utilities.proc. VectorComparison 
  
   getProteins(Node)   - 
Method in class sbrt.shell.text.fba. PalssonRxnNodeParser 
 Calls  PalssonRxnNodeParser.getCatalysts(Node, String)  with " PROTEIN_ASSOCIATION: "
 as the  prefix .
   getRangeCutoff()   - 
Method in exception sbrt.kernel.fba.comp. FluxRangeException 
 Returns the defined range cutoff.
   getRatio(LinearComb<?>, LinearComb<?>)   - 
Method in class sbrt.kernel.fba. AbstractRxn 
 Returns the ratio of the coefficients in the provided expressions.
   getReactants()   - 
Method in class sbrt.kernel.fba. AbstractRxn 
 Returns the reactants of this reaction.
   getReactants()   - 
Method in interface sbrt.kernel.fba. ChemRxn 
 Returns the reactants of this chemical reaction as a 
 linear combination of chemical species.
   getReactants()   - 
Method in class sbrt.kernel.fba. ExchangeRxn 
 Returns an empty linear combination.
   getReactants()   - 
Method in class sbrt.kernel.fba. SinkRxn 
 Returns a single-term linear combination.
   getReactants(Node)   - 
Method in class sbrt.shell.text.fba. BiggRxnNodeParser 
 Returns the reactants of the provided reaction node.
   getReactants(Node)   - 
Method in class sbrt.shell.text.fba. PalssonRxnNodeParser 
 Returns the reactants of the provided reaction node.
   getReactioName()   - 
Method in exception sbrt.kernel.fba.comp. FluxCapException 
 Returns the name of the reaction whose flux was intended to be capped.
   getRegex()   - 
Method in class sbrt.shell.text. AbstractLinkedHashSetFormat 
 Returns the regular expression used to separate
 elements when parsing a formatted string.
   getRegex()   - 
Method in class sbrt.shell.text. AbstractListFormat 
 Returns the regular expression used to separate elements when parsing a
 formatted string.
   getRequiredKeys()   - 
Method in class sbrt.shell.io. AbstractMapInputFile 
 Returns the set of keys that must be contained in this
 file.
   getRequiredKeys()   - 
Method in class sbrt.shell.mng.algebra. LinearCombConverterManager 
 Returns the set of keywords required by this process manager.
   getRequiredKeys()   - 
Method in class sbrt.shell.mng.algebra. LinearSolverManager 
 Returns the set of keywords required by this process manager.
   getRequiredKeys()   - 
Method in class sbrt.shell.mng.algebra. MatrixConverterManager 
 Returns the set of keywords required by this process manager.
   getRequiredKeys()   - 
Method in class sbrt.shell.mng.algebra. MultipleVectorsConverterManager 
 Returns the set of keywords required by this process manager.
   getRequiredKeys()   - 
Method in class sbrt.shell.mng.algebra. SingleVectorConverterManager 
 Returns the set of keywords required by this process manager.
   getRequiredKeys()   - 
Method in class sbrt.shell.mng.comb. SingleElementUnionManager 
 Returns the set of keywords required by this process manager.
   getRequiredKeys()   - 
Method in class sbrt.shell.mng.comb. StrictSingleElementUnionManager 
 Returns the set of keywords required by this process manager.
   getRequiredKeys()   - 
Method in class sbrt.shell.mng.fba. BiggSbmlReaderManager 
 Returns the set of keywords required by this process manager.
   getRequiredKeys()   - 
Method in class sbrt.shell.mng.fba. BiggSbmlTranslaterManager 
 Returns the set of keywords required by this process manager.
   getRequiredKeys()   - 
Method in class sbrt.shell.mng.fba. CatalystDeletionManager 
 Returns the set of keywords the provided  ManagerInput  object must
 contain.
   getRequiredKeys()   - 
Method in class sbrt.shell.mng.fba. CdHarFluxVectorManager 
 Returns the set of keywords the provided  ManagerInput  object must
 contain.
   getRequiredKeys()   - 
Method in class sbrt.shell.mng.fba. CvCatalystDeletionManager 
 Returns the set of keywords the provided  ManagerInput  object must
 contain.
   getRequiredKeys()   - 
Method in class sbrt.shell.mng.fba. CvFbaOptManager 
 Returns the set of keywords the provided 
  ManagerInput  object must contain.
   getRequiredKeys()   - 
Method in class sbrt.shell.mng.fba. CvMsFluxIntervalManager 
 Returns the set of keywords the provided 
  ManagerInput  object must contain.
   getRequiredKeys()   - 
Method in class sbrt.shell.mng.fba. CvObjectiveFunctionManager 
 Returns the set of keywords the provided 
  ManagerInput  object must contain.
   getRequiredKeys()   - 
Method in class sbrt.shell.mng.fba. CvRevRxnFluxIntervalManager 
 Returns the set of keywords the provided  ManagerInput  object must
 contain.
   getRequiredKeys()   - 
Method in class sbrt.shell.mng.fba. CvRxnDeletionManager 
 Returns the set of keywords the provided 
  ManagerInput  object must contain.
   getRequiredKeys()   - 
Method in class sbrt.shell.mng.fba. CvSimpleFluxIntervalManager 
 Returns the set of keywords the provided 
  ManagerInput  object must contain.
   getRequiredKeys()   - 
Method in class sbrt.shell.mng.fba. ExtremeCurrentManager 
 Returns the set of keywords the provided 
  ManagerInput  object must contain.
   getRequiredKeys()   - 
Method in class sbrt.shell.mng.fba. FbaOptManager 
 Returns the set of keywords the provided  ManagerInput  object must
 contain.
   getRequiredKeys()   - 
Method in class sbrt.shell.mng.fba. FbaSolverManager 
 Returns the set of keywords the provided 
  ManagerInput  object must contain.
   getRequiredKeys()   - 
Method in class sbrt.shell.mng.fba. FluxActivityManager 
 Returns the set of keywords the provided  ManagerInput  object must
 contain.
   getRequiredKeys()   - 
Method in class sbrt.shell.mng.fba. FluxCapManager 
 Returns the set of keywords the provided 
  ManagerInput  object must contain.
   getRequiredKeys()   - 
Method in class sbrt.shell.mng.fba. FluxPlasticityManager 
 Returns the set of keywords the provided  ManagerInput  object must
 contain.
   getRequiredKeys()   - 
Method in class sbrt.shell.mng.fba. InitialPointManager 
 Returns the set of keywords the provided  ManagerInput  object must
 contain.
   getRequiredKeys()   - 
Method in class sbrt.shell.mng.fba. MetatoolFileWriterManager 
 Returns the set of keywords required by this process manager.
   getRequiredKeys()   - 
Method in class sbrt.shell.mng.fba. MmaNotebookManager 
 Returns the set of keywords the provided  ManagerInput  object must
 contain.
   getRequiredKeys()   - 
Method in class sbrt.shell.mng.fba. MsFluxIntervalManager 
 Returns the set of keywords the provided  ManagerInput  object must
 contain.
   getRequiredKeys()   - 
Method in class sbrt.shell.mng.fba. MsFluxomeReducerManager 
 Returns the set of keywords the provided 
  ManagerInput  object must contain.
   getRequiredKeys()   - 
Method in class sbrt.shell.mng.fba. NetworkInfoManager 
 Returns the set of keywords the provided  ManagerInput  object must
 contain.
   getRequiredKeys()   - 
Method in class sbrt.shell.mng.fba. ObjectiveFunctionManager 
 Returns the set of keywords the provided 
  ManagerInput  object must contain.
   getRequiredKeys()   - 
Method in class sbrt.shell.mng.fba. PalssonSbmlReaderManager 
 Returns the set of keywords required by this process manager.
   getRequiredKeys()   - 
Method in class sbrt.shell.mng.fba. PalssonSbmlTranslaterManager 
 Returns the set of keywords required by this process manager.
   getRequiredKeys()   - 
Method in class sbrt.shell.mng.fba. RandomConstraintsManager 
 Returns the set of keywords the provided 
  ManagerInput  object must contain.
   getRequiredKeys()   - 
Method in class sbrt.shell.mng.fba. RandomEnvManager 
 Returns the set of keywords the provided  ManagerInput  object must
 contain.
   getRequiredKeys()   - 
Method in class sbrt.shell.mng.fba. RandomObjectiveManager 
 Returns the set of keywords the provided 
  ManagerInput  object must contain.
   getRequiredKeys()   - 
Method in class sbrt.shell.mng.fba. ReverseRxnFluxIntervalManager 
 Returns the set of keywords the provided  ManagerInput  object must
 contain.
   getRequiredKeys()   - 
Method in class sbrt.shell.mng.fba. RxnDeletionManager 
 Returns the set of keywords the provided 
  ManagerInput  object must contain.
   getRequiredKeys()   - 
Method in class sbrt.shell.mng.fba. RxnRemovalManager 
 Returns the set of keywords the provided  ManagerInput  object must
 contain.
   getRequiredKeys()   - 
Method in class sbrt.shell.mng.fba. SbmlWriterManager 
 Returns the set of keywords required by this process manager.
   getRequiredKeys()   - 
Method in class sbrt.shell.mng.fba. SimpleFluxIntervalManager 
 Returns the set of keywords the provided  ManagerInput  object must
 contain.
   getRequiredKeys()   - 
Method in class sbrt.shell.mng.fba. SimpleRxnFileTransManager 
 Returns the set of keywords required by this process manager.
   getRequiredKeys()   - 
Method in class sbrt.shell.mng.fba. SlpCycleIdManager 
 Returns the set of keywords the provided  ManagerInput  object must
 contain.
   getRequiredKeys()   - 
Method in class sbrt.shell.mng.fba. StoichVariationManager 
 Returns the set of keywords the provided  ManagerInput  object must
 contain.
   getRequiredKeys()   - 
Method in class sbrt.shell.mng.fba. SvCdHarFluxVectorManager 
 Returns the set of keywords the provided  ManagerInput  object must
 contain.
   getRequiredKeys()   - 
Method in class sbrt.shell.mng.fba. SvInitialPointManager 
 Returns the set of keywords the provided  ManagerInput  object must
 contain.
   getRequiredKeys()   - 
Method in class sbrt.shell.mng.fba. WwFluxomeReducerManager 
 Returns the set of keywords the provided 
  ManagerInput  object must contain.
   getRequiredKeys()   - 
Method in class sbrt.shell.mng.geom. CdHarManager 
 Returns the set of keywords required by this process manager.
   getRequiredKeys()   - 
Method in class sbrt.shell.mng.graph_theory. CycleIdManager 
 Returns the set of keywords the provided 
  ManagerInput  object must contain.
   getRequiredKeys()   - 
Method in class sbrt.shell.mng.graph_theory. PathIdManager 
 Returns the set of keywords the provided 
  ManagerInput  object must contain.
   getRequiredKeys()   - 
Method in class sbrt.shell.mng.graph_theory. UniqueCycleIdManager 
 Returns the set of keywords the provided 
  ManagerInput  object must contain.
   getRequiredKeys()   - 
Method in interface sbrt.shell.mng. ProcessManager 
 Returns the set of keys the provided
  ManagerInput  object must contain.
   getRequiredKeys()   - 
Method in class sbrt.shell.mng.stat. CorrelationManager 
 Returns the set of keywords required by this process manager.
   getRequiredKeys()   - 
Method in class sbrt.shell.mng.stat. KendallTauManager 
 Returns the set of keywords required by this process manager.
   getRequiredKeys()   - 
Method in class sbrt.shell.mng.stat. MannWhitneyUManager 
 Returns the set of keywords required by this process manager.
   getRequiredKeys()   - 
Method in class sbrt.shell.mng.utilities. IntervalComparisonManager 
 Returns the set of keywords required by this process manager.
   getRequiredKeys()   - 
Method in class sbrt.shell.mng.utilities. VariableParticipationManager 
 Returns the set of keywords the provided 
  ManagerInput  object must contain.
   getRequiredKeys()   - 
Method in class sbrt.shell.mng.utilities. VectorComparisonManager 
 Returns the set of keywords required by this process manager.
   getRequiredValue(Object)   - 
Method in class sbrt.shell.io. ProcessInputFile 
 Returns the value corresponding to the provided key.
   getRequiredValue(Object)   - 
Method in interface sbrt.shell.mng. ManagerInput 
 Returns the value corresponding to the provided key.
   getRequiredValue(Object)   - 
Method in class sbrt.shell.mng. MapManagerInput 
 Returns the value corresponding to the provided key.
   getResults()   - 
Method in class sbrt.kernel.io. ListResultsManager 
 Returns a copy of the list of results added to this results manager.
   getResults()   - 
Method in class sbrt.kernel.io. SingleRm 
 Returns the last result added to this results manager.
   getReverseRxnNames(Collection<String>)   - 
Method in class sbrt.kernel.fba.comp. AbstractFluxIntervalComputer 
 Returns the name of each provided reaction mapped to the names of 
 each of its reverse reactions.
   getReverseRxnNames(String)   - 
Method in interface sbrt.kernel.fba. Fluxome 
 Returns the set of names of reactions in this fluxome that are
 stoichiometrically equivalent to the provided reaction in reverse.
   getReverseRxnNames(String)   - 
Method in class sbrt.kernel.fba. IrrevRxnFluxomeV1 
 Returns the names of all of the reactions in this fluxome that are
 stoichiometrically equivalent to the provided reaction in reverse.
   getReverseRxns(String)   - 
Method in interface sbrt.kernel.fba. IrrevRxnFluxome 
 Returns the set reactions in this fluxome that are
 stoichiometrically equivalent to the provided reaction in reverse.
   getReverseRxns(String)   - 
Method in class sbrt.kernel.fba. IrrevRxnFluxomeV1 
 Returns all of the reactions in this fluxome that are
 stoichiometrically equivalent to the provided reaction in reverse.
   getRightPadding()   - 
Method in class sbrt.shell.text. AbstractMapEntryFormat 
 Returns the right padding used to create formatted
 string representations of key-value pairs.
   getRow(Object)   - 
Method in interface sbrt.kernel.math.algebra. DoubleMatrix 
 Returns the row corresponding to the provided row index.
   getRow(Object)   - 
Method in class sbrt.kernel.math.algebra. DoubleMatrixWrap 
 Returns the row corresponding to the provided row index.
   getRow(Object)   - 
Method in class sbrt.kernel.math.algebra. HashMatrix 
 Returns the row corresponding to the provided row index.
   getRow(Object)   - 
Method in interface sbrt.kernel.math.algebra. Matrix 
 Returns the row corresponding to the provided row index.
   getRow(Object)   - 
Method in class sbrt.kernel.math.algebra. MatrixWrap 
 Returns the row corresponding to the provided row index.
   getRow(Object)   - 
Method in class sbrt.kernel.math.algebra. RowOrTreeMatrix 
 Returns the row corresponding to the provided row index.
   getRow(Object)   - 
Method in interface sbrt.kernel.math.algebra. SparseDoubleMatrix 
 Returns the row corresponding to the provided row index.
   getRow(Object)   - 
Method in class sbrt.kernel.math.algebra. SparseDoubleMatrixWrap 
 Returns the row corresponding to the provided row index.
   getRow(Object)   - 
Method in class sbrt.kernel.math.algebra. TreeMatrix 
 Returns the row corresponding to the provided row index.
   getRowConstraint(Object)   - 
Method in class sbrt.kernel.fba.comp. FbaOptComputer 
 Returns the current constraint on the provided row variable.
   getRowConstraints()   - 
Method in class sbrt.kernel.fba.comp. FbaOptComputer 
 Returns all the current constraints on row variables.
   getRowHeaders()   - 
Method in class sbrt.shell.io.fba. RowOrFbaOptOutputFile 
 Returns the set of row headers whose values will be
 written to this output file when results are added.
   getRowIndex()   - 
Method in class sbrt.kernel.math.algebra. IndexPair 
 Returns the row index.
   getRowIndex()   - 
Method in class sbrt.kernel.math.algebra. MatrixElement 
 Returns the row index of this matrix element.
   getRowIndexFormat()   - 
Method in class sbrt.shell.text.math. IndexPairFormatV1 
 Returns the format used for row indices.
   getRowIndices()   - 
Method in class sbrt.kernel.math.algebra. HashMatrix 
 Returns the set of row indices contained in this
 matrix.
   getRowIndices()   - 
Method in interface sbrt.kernel.math.algebra. Matrix 
 Returns the set of row indices contained in this
 matrix.
   getRowIndices()   - 
Method in class sbrt.kernel.math.algebra. MatrixWrap 
 Returns the set of row indices contained in this
 matrix.
   getRowIndices()   - 
Method in class sbrt.kernel.math.algebra. RowOrTreeMatrix 
 Returns the set of row indices contained in this
 matrix.
   getRowIndices()   - 
Method in class sbrt.kernel.math.algebra. TreeMatrix 
 Returns the set of row indices contained in this
 matrix.
   getRowIndNzEl(Object)   - 
Method in class sbrt.kernel.math.algebra. HashMatrix 
 Returns the set of row indices whose elements are nonzero 
 in the column corresponding to the provided index.
   getRowIndNzEl(Object)   - 
Method in class sbrt.kernel.math.algebra. RowOrTreeMatrix 
 Returns the set of row indices whose elements are nonzero 
 in the column corresponding to the provided index.
   getRowIndNzEl(Object)   - 
Method in interface sbrt.kernel.math.algebra. SparseDoubleMatrix 
 Returns the set of row indices whose elements are nonzero 
 in the column corresponding to the provided index.
   getRowIndNzEl(Object)   - 
Method in class sbrt.kernel.math.algebra. SparseDoubleMatrixWrap 
 Returns the set of row indices whose elements are nonzero 
 in the column corresponding to the provided index.
   getRowIndNzEl(Object)   - 
Method in class sbrt.kernel.math.algebra. TreeMatrix 
 Returns the set of row indices whose elements are nonzero 
 in the column corresponding to the provided index.
   getRowVariables()   - 
Method in class sbrt.kernel.fba.comp. FbaOptComputer 
 Returns the set of all row variables in the program matrix.
   getRxn(String)   - 
Method in interface sbrt.kernel.fba. Fluxome 
 Returns the chemical reaction in this fluxome with the provided name.
   getRxn(String)   - 
Method in class sbrt.kernel.fba. IrrevRxnFluxomeV1 
 Returns the chemical reaction in this fluxome with the provided name.
   getRxnExpr(String)   - 
Method in class sbrt.kernel.fba.comp. AbstractCvComputer 
 Converts a reaction name to a single-term linear combination.
   getRxnExpr(Collection<String>)   - 
Method in class sbrt.kernel.fba.comp. AbstractCvComputer 
 Converts a collection of reaction names to a linear combination.
   getRxnExpr(String)   - 
Method in class sbrt.kernel.fba.comp. FbaOptComputer 
 Converts a reaction name to a single-term linear combination.
   getRxnExpr(Collection<String>)   - 
Method in class sbrt.kernel.fba.comp. FbaOptComputer 
 Converts a collection of reaction names to a linear combination.
   getRxnExprConstraint(MathExpr<String>)   - 
Method in class sbrt.kernel.fba.comp. AbstractFluxIntervalComputer 
 Returns the current constraint on the provided expression.
   getRxnExprConstraint(MathExpr<String>)   - 
Method in class sbrt.kernel.fba.comp. CdHarFluxVectorComputer 
 Returns the current constraint on the provided expression.
   getRxnExprConstraint(MathExpr<String>)   - 
Method in class sbrt.kernel.fba.comp. FastMsFluxRangeComputer 
 Returns the current constraint on the provided expression.
   getRxnExprConstraint(MathExpr<String>)   - 
Method in class sbrt.kernel.fba.comp. FbaOptComputer 
 Returns the current constraint on the provided expression.
   getRxnExprConstraint(MathExpr<String>)   - 
Method in interface sbrt.kernel.fba.comp. FluxCvComputer 
 Returns the current constraint on the provided expression.
   getRxnExprConstraint(MathExpr<String>)   - 
Method in class sbrt.kernel.fba.comp. InitialPointComputer 
 Returns the current constraint on the provided expression.
   getRxnExprConstraint(MathExpr<String>)   - 
Method in class sbrt.kernel.fba.comp. RxnDeletionComputer 
 Returns the constraint on the provided mathematical expression of
 fluxes.
   getRxnExprConstraint(MathExpr<String>)   - 
Method in interface sbrt.kernel.fba. Fluxome 
 Returns the constraint corresponding to the provided
 mathematical expression of reaction names.
   getRxnExprConstraint(MathExpr<String>)   - 
Method in class sbrt.kernel.fba. IrrevRxnFluxomeV1 
 Returns the constraint associated with the expression provided.
   getRxnExprConstraints()   - 
Method in class sbrt.kernel.fba.comp. AbstractFluxIntervalComputer 
 Returns all current flux expression constraints.
   getRxnExprConstraints()   - 
Method in class sbrt.kernel.fba.comp. CdHarFluxVectorComputer 
 Returns all current flux expression constraints.
   getRxnExprConstraints()   - 
Method in class sbrt.kernel.fba.comp. FastMsFluxRangeComputer 
 Returns all current flux expression constraints.
   getRxnExprConstraints()   - 
Method in class sbrt.kernel.fba.comp. FbaOptComputer 
 Returns all current constraints on mathematical expressions
 of reaction names.
   getRxnExprConstraints()   - 
Method in interface sbrt.kernel.fba.comp. FluxCvComputer 
 Returns all current flux expression constraints.
   getRxnExprConstraints()   - 
Method in class sbrt.kernel.fba.comp. InitialPointComputer 
 Returns all current flux expression constraints.
   getRxnExprConstraints()   - 
Method in class sbrt.kernel.fba.comp. RxnDeletionComputer 
 Returns the set of all constraints on mathematical expressions of
 fluxes.
   getRxnExprs()   - 
Method in class sbrt.kernel.fba.comp. FbaOptComputer 
 Returns the set of all mathematical expressions of reaction names.
   getRxnFormat()   - 
Method in class sbrt.shell.text.fba. IrfFormatV1 
 Returns the format used for reactions.
   getRxnId(Node)   - 
Method in class sbrt.shell.text.fba. BiggRxnNodeParser 
 Returns the reaction ID of the provided reaction node.
   getRxnId(Node)   - 
Method in class sbrt.shell.text.fba. PalssonRxnNodeParser 
 Returns the reaction ID of the provided reaction node.
   getRxnNameChecker()   - 
Method in class sbrt.shell.text.fba. RxnNameExprFormatV1 
 Returns the reaction name verifier.
   getRxnNameChecker()   - 
Method in class sbrt.shell.text.fba. RxnNameListFormatV1 
 Returns the reaction name verifier.
   getRxnNameChecker()   - 
Method in class sbrt.shell.text.fba. RxnNameOrExprFormatV1 
 Returns the reaction name verifier.
   getRxnNameChecker()   - 
Method in class sbrt.shell.text.fba. RxnNameSetFormatV1 
 Returns the reaction name verifier.
   getRxnNameExprFormat(Fluxome)   - 
Static method in class sbrt.shell.mng.fba. FbaManager 
 Returns the default reaction name expression format.
   getRxnNameExprSet()   - 
Method in class sbrt.shell.io.fba. RxnNamExprSetInputFile 
 Returns the set of reaction name expressions contained in this fle.
   getRxnNameFormat()   - 
Method in class sbrt.shell.text.fba. IrfFormatV1 
 Returns the format used for reaction names.
   getRxnNameHeaders(ManagerInput<String, String>, Fluxome)   - 
Static method in class sbrt.shell.mng.fba. FbaManager 
 Parses and returns the value for   AbstractProcessManager.DATA_HEADERS   in the 
 provided manager input.
   getRxnNameHeaders(String, Fluxome)   - 
Static method in class sbrt.shell.mng.fba. FbaManager 
 Parses and returns the set of reaction names contained in the provided
 string.
   getRxnNames(String)   - 
Method in interface sbrt.kernel.fba. CatalyzedFluxome 
 Returns the set of names of all chemical reactions 
 contained in this fluxome that are catalyzed by the provided
 catalyst.
   getRxnNames()   - 
Method in interface sbrt.kernel.fba. Fluxome 
 Returns the set of names of all chemical reactions 
 contained in this fluxome.
   getRxnNames(String)   - 
Method in class sbrt.kernel.fba. IrrevRxnFluxomeV1 
 Returns the names of the reactions catalyzed by the provided catalyst.
   getRxnNames()   - 
Method in class sbrt.kernel.fba. IrrevRxnFluxomeV1 
 Returns the set of names of all chemical reactions 
 contained in this fluxome.
   getRxnNames(Set<String>, IrrevRxnFluxome)   - 
Static method in class sbrt.kernel.fba.proc. CatalystDeletionProcess 
 Returns the set of names of reactions whose activity depends on the
 provided set of catalysts.
   getRxnNames()   - 
Method in class sbrt.kernel.fba.proc. NetworkInfoProcess.Results 
 Returns the names of all reactions contained in the fluxome.
   getRxnNameSetFormat()   - 
Method in class sbrt.shell.text.fba. FbaOptHeaderFormatterV1 
 Returns the format used for reaction name sets.
   getRxnNameSetFormat()   - 
Method in class sbrt.shell.text.fba. FbaOptHeaderParserV1 
 Returns the format used for reaction name sets.
   getRxns(String)   - 
Method in interface sbrt.kernel.fba. CatalyzedFluxome 
 Returns the chemical reactions contained in this fluxome
 that are catalyzed by the provided catalyst.
   getRxns()   - 
Method in interface sbrt.kernel.fba. Fluxome 
 Returns the set of all chemical reactions contained in this fluxome.
   getRxns(Collection<String>)   - 
Method in interface sbrt.kernel.fba. Fluxome 
 Returns the chemical reactions with the provided names.
   getRxns(String)   - 
Method in class sbrt.kernel.fba. IrrevRxnFluxomeV1 
 Returns all of the internal reactions contained in this fluxome
 that are catalyzed by the provided catalyst.
   getRxns()   - 
Method in class sbrt.kernel.fba. IrrevRxnFluxomeV1 
 Returns all chemical reactions contained in this fluxome.
   getRxns(Collection<String>)   - 
Method in class sbrt.kernel.fba. IrrevRxnFluxomeV1 
 Returns the chemical reactions with the provided names.
   getS(Map<? extends V, Double>, Map<? extends V, Double>)   - 
Method in class sbrt.kernel.math.stat.comp. KendallTau 
 Computes and returns the S value of the provided values.
   getS(Collection<Double>, Collection<Double>)   - 
Method in class sbrt.kernel.math.stat.comp. KendallTau 
 Computes and returns the S value of the provided values.
   getS(double[], double[])   - 
Method in class sbrt.kernel.math.stat.comp. KendallTau 
 Computes and returns the S value of the provided values.
   getSafetyLevel(ManagerInput<String, String>, PrintWriter)   - 
Static method in class sbrt.shell.mng.fba. FbaOptManager 
 Parses and returns the value for   FbaOptManager.SAFETY_LEVEL   in the provided
 manager input.
   getSample(String, PrintWriter)   - 
Static method in class sbrt.shell.mng.stat. MannWhitneyUManager 
 Returns the list of values contained in the file with the provided name.
   getSample(String)   - 
Static method in class sbrt.shell.mng.stat. MannWhitneyUManager 
 Returns the list of values contained in the file with the provided name.
   getSeed(ManagerInput<String, String>, PrintWriter)   - 
Static method in class sbrt.shell.mng. AbstractProcessManager 
 Returns the parsed value for   AbstractProcessManager.SEED   in the provided input and
 prints a descriptive message to the provided print writer.
   getSense()   - 
Method in exception sbrt.kernel.fba.comp. FbaOptException 
 Returns the sense, or direction, of the attempted optimization.
   getSet()   - 
Static method in class sbrt.shell.text. SpecialChars 
 Returns a set of the characters contained in this class.
   getSetFormat()   - 
Method in class sbrt.shell.text. AbstractMapFormat 
 Returns the format used for sets of keys.
   getSetFormat()   - 
Method in class sbrt.shell.text. AppInputFileLineFormatVersion1 
 Returns the format used for sets of variables.
   getSetFormat()   - 
Method in class sbrt.shell.text. ColonMapFormatV1 
 Returns the format used for sets of variables.
   getSetFormat()   - 
Method in class sbrt.shell.text. EqualsMapFormatV1 
 Returns the format used for sets of variables.
   getSetFormat()   - 
Method in class sbrt.shell.text.fba. CatalystSetFormatV1 
 Returns the format used for sets of catalyst names.
   getSetFormat()   - 
Method in class sbrt.shell.text.fba. ConstraintsFileLineFormatV1 
 Returns the format used for sets of variables.
   getSetFormat()   - 
Method in class sbrt.shell.text.fba. ConstraintsFileLineFormatV2 
 Returns the format used for sets of variables.
   getSetFormat()   - 
Method in class sbrt.shell.text.fba. FluxCapFormatV1 
 Returns the format used for sets of variables.
   getSetFormat()   - 
Method in class sbrt.shell.text.fba. FluxomeSolFileLineFormatV1 
 Returns the format used for sets of variables.
   getSetFormat()   - 
Method in class sbrt.shell.text.fba. FluxVectorFormatV1 
 Returns the format used for sets of variables.
   getSetFormat()   - 
Method in class sbrt.shell.text.fba. IrfFormatV1 
 Returns the format used for sets of reaction names.
   getSetFormat()   - 
Method in class sbrt.shell.text.fba. RxnNameSetFormatV1 
 Returns the format used for sets of reaction names.
   getSetFormat()   - 
Method in class sbrt.shell.text. SimpleSetFormat 
 Returns the underlying set format.
   getSets(String, PrintWriter)   - 
Static method in class sbrt.shell.mng.comb. SingleElementUnionManager 
 Returns the list of sets contained in the file with the provided name.
   getSets(String)   - 
Static method in class sbrt.shell.mng.comb. SingleElementUnionManager 
 Returns the list of sets contained in the file with the provided name.
   getSets(ManagerInput<String, String>, PrintWriter)   - 
Static method in class sbrt.shell.mng.comb. SingleElementUnionManager 
 Returns the list of sets contained in the file with the name contained in the
 provided input.
   getSingleFluxVectorOutputFile(ManagerInput<String, String>, PrintWriter, Fluxome)   - 
Static method in class sbrt.shell.mng.fba. FbaManager 
 Returns an empty output file that is suitable for storing a single
 flux vector.
   getSingleFluxVectorOutputFile(String, FileFormat, Set<String>, Fluxome)   - 
Static method in class sbrt.shell.mng.fba. FbaManager 
 Returns an empty output file that is suitable for storing a single
 flux vector.
   getSingleResultOutputFile(ManagerInput<String, String>, PrintWriter, Fluxome)   - 
Static method in class sbrt.shell.mng.fba. FbaOptManager 
 Returns an empty output file that is suitable for storing a single FBA
 optimization result.
   getSingleResultOutputFile(String, FileFormat, Set<String>, Fluxome)   - 
Static method in class sbrt.shell.mng.fba. FbaOptManager 
 Returns an empty output file that is suitable for storing a single FBA
 optimization result.
   getSingleResultOutputFile(ManagerInput<String, String>, PrintWriter, Fluxome)   - 
Static method in class sbrt.shell.mng.fba. SimpleFluxIntervalManager 
 Returns an empty output file that is suitable for storing a single set of
 flux interval results.
   getSingleResultOutputFile(String, Set<String>, Fluxome)   - 
Static method in class sbrt.shell.mng.fba. SimpleFluxIntervalManager 
 Returns an empty output file that is suitable for storing a single set of
 flux interval results.
   getSingletonSets(Set<E>)   - 
Method in class sbrt.kernel.math.comb.comp. SingleElementUnionsV1 
 Returns a set of singleton sets created from the provided set.
   getSingletonSets(Set<E>)   - 
Method in class sbrt.kernel.math.comb.comp. SingleElementUnionsV2 
 Returns a set of singleton sets created from the provided set.
   getSingletonSets(Set<E>)   - 
Method in class sbrt.kernel.math.comb.comp. StrictSingleElementUnions 
 Returns a set of singleton sets created from the provided set.
   getSink()   - 
Method in class sbrt.kernel.math.graph_theory. DirectedGraph.Edge 
 Returns the sink node of this directed edge.
   getSinkRxn(String)   - 
Method in interface sbrt.kernel.fba. IrrevRxnFluxome 
 Returns the sink reaction in this fluxome with the provided name.
   getSinkRxn(String)   - 
Method in class sbrt.kernel.fba. IrrevRxnFluxomeV1 
 Returns the sink reaction in this fluxome with the provided name.
   getSinkRxnNames()   - 
Method in interface sbrt.kernel.fba. IrrevRxnFluxome 
 Returns the set of names of all sink reactions in this fluxome.
   getSinkRxnNames()   - 
Method in class sbrt.kernel.fba. IrrevRxnFluxomeV1 
 Returns the set of names of all sinks in this fluxome.
   getSinkRxns(Collection<String>)   - 
Method in interface sbrt.kernel.fba. IrrevRxnFluxome 
 Returns the sink reactions with the provided names.
   getSinkRxns()   - 
Method in interface sbrt.kernel.fba. IrrevRxnFluxome 
 Returns the set of all sink reactions in this fluxome.
   getSinkRxns()   - 
Method in class sbrt.kernel.fba. IrrevRxnFluxomeV1 
 Returns all of the sinks in this fluxome.
   getSinkRxns(Collection<String>)   - 
Method in class sbrt.kernel.fba. IrrevRxnFluxomeV1 
 Returns the sink reactions whose names appear in the provided
 collection of reaction names.
   getSmallest(double, double)   - 
Static method in class sbrt.kernel.util. Util 
 Returns the smallest of the provided numbers.
   getSmallest(int, int)   - 
Static method in class sbrt.kernel.util. Util 
 Returns the smallest of the provided numbers.
   getSolution(Object)   - 
Method in class sbrt.kernel.math.algebra. HashLinearSystemSolution 
 Returns the linear combination of free variables to which the provided
 variable is equal.
   getSolution(Object)   - 
Method in interface sbrt.kernel.math.algebra. LinearSystemSolution 
 Returns the linear combination of free variables to which the provided
 variable is equal.
   getSolutionVector()   - 
Method in class sbrt.ext.cplex. CplexSolver 
 Returns the most recently computed solution vector.
   getSolutionVector()   - 
Method in class sbrt.ext.glpk. GlpkSolver 
 Returns the most recently computed solution vector.
   getSolutionVector()   - 
Method in class sbrt.kernel.fba.comp. FbaOptComputer.Results 
 Returns the solution vector computed during optimization.
   getSolutionVector(Map<?, Double>)   - 
Method in class sbrt.kernel.math.algebra. HashLinearSystemSolution 
 Computes and returns a solution vector by plugging in the provided values
 for the free variables in this solution.
   getSolutionVector(Map<?, Double>)   - 
Method in interface sbrt.kernel.math.algebra. LinearSystemSolution 
 Computes and returns a solution vector by plugging in the 
 provided values for the free variables in this solution.
   getSolutionVector()   - 
Method in interface sbrt.kernel.math.optimization. ProgramSolver 
 Returns the most recently computed solution vector.
   getSolver(ManagerInput<String, String>, PrintWriter)   - 
Static method in class sbrt.shell.mng.algebra. LinearSolverManager 
 Returns the linear system solver specified by   LinearSolverManager.KERNEL_LINK_CMD  
 in the provided input.
   getSorted(int[])   - 
Static method in class sbrt.kernel.util. Util 
 Returns a new array containing the values from the provided array
 in ascending numerical order.
   getSorted(double[])   - 
Static method in class sbrt.kernel.util. Util 
 Returns a new array containing the values from the provided array
 in ascending numerical order.
   getSortedArray(Collection<? extends Number>)   - 
Static method in class sbrt.kernel.util. Util 
 Returns a sorted array containing the values in the provided collection.
   getSortedArray(Collection<Integer>)   - 
Static method in class sbrt.kernel.util. Util 
 Returns a sorted array containing the values in the provided collection.
   getSource()   - 
Method in class sbrt.kernel.math.graph_theory. DirectedGraph.Edge 
 Returns the source node of this directed edge.
   getSourceRxn(String)   - 
Method in interface sbrt.kernel.fba. IrrevRxnFluxome 
 Returns the source reaction in this fluxome with the provided name.
   getSourceRxn(String)   - 
Method in class sbrt.kernel.fba. IrrevRxnFluxomeV1 
 Returns the source reaction in this fluxome with the provided name.
   getSourceRxnNames()   - 
Method in interface sbrt.kernel.fba. IrrevRxnFluxome 
 Returns the set of names of all source reactions in this fluxome.
   getSourceRxnNames()   - 
Method in class sbrt.kernel.fba. IrrevRxnFluxomeV1 
 Returns the set of names of all sources in this fluxome.
   getSourceRxns(Collection<String>)   - 
Method in interface sbrt.kernel.fba. IrrevRxnFluxome 
 Returns the source reactions with the provided names.
   getSourceRxns()   - 
Method in interface sbrt.kernel.fba. IrrevRxnFluxome 
 Returns the set of all source reactions in this fluxome.
   getSourceRxns()   - 
Method in class sbrt.kernel.fba. IrrevRxnFluxomeV1 
 Returns all of the sources in this fluxome.
   getSourceRxns(Collection<String>)   - 
Method in class sbrt.kernel.fba. IrrevRxnFluxomeV1 
 Returns the source reactions whose names appear in the provided
 collection of reaction names.
   getSpecies()   - 
Method in class sbrt.kernel.fba. ExchangeRxn 
 Returns the chemical species associated with this exchange reaction.
   getSpecies()   - 
Method in interface sbrt.kernel.fba. Fluxome 
 Returns the set of names of all chemical species participating
 in reactions contained in this fluxome.
   getSpecies()   - 
Method in class sbrt.kernel.fba. IrrevRxnFluxomeV1 
 Returns the set of names of all chemical species participating
 in reactions contained in this fluxome.
   getStatistic()   - 
Method in class sbrt.ext.R.stat. RCorrelator.Result 
 Returns the corresponding statistic.
   getStatus()   - 
Method in class sbrt.ext.cplex. CplexSolver 
 Returns the status of the most recently attempted optimization.
   getStatus()   - 
Method in class sbrt.ext.glpk. GlpkSolver 
 Returns the status of the most recently attempted optimization.
   getStatus()   - 
Method in exception sbrt.kernel.fba.comp. FbaOptException 
 Returns the status of the attempted optimization.
   getStatus()   - 
Method in interface sbrt.kernel.math.optimization. ProgramSolver 
 Returns the status of the most recently attempted optimization.
   getStdev()   - 
Method in class sbrt.kernel.math.stat. NormalDist 
 Returns the standard deviation of this distribution.
   getStoichiometryMatrix()   - 
Method in interface sbrt.kernel.fba. Fluxome 
 Returns the stoichiometry matrix of this fluxome.
   getStoichiometryMatrix()   - 
Method in class sbrt.kernel.fba. IrrevRxnFluxomeV1 
 Returns the stoichiometry matrix of this fluxome.
   getStoichMatrix()   - 
Method in class sbrt.kernel.fba.proc. NetworkInfoProcess.Results 
 Returns the stoichiometry matrix of the fluxome.
   getSuccessors(Object)   - 
Method in class sbrt.kernel.math.graph_theory. DirectedGraph 
 Returns the successors of the provided node.
   getSvInputFile(ManagerInput<String, String>, PrintWriter, Fluxome)   - 
Static method in class sbrt.shell.mng.fba. IntervalVariationManager 
 Returns the space variation input with the name denoted by
   IntervalVariationManager.FLUX_INTERVAL_FILE   in the provided manager input.
   getSystemSolution()   - 
Method in class sbrt.kernel.math.geom. HashConvexPolytopeOld 
 Returns the linear system solution used to form this convex polytope.
   getT(double, int, int)   - 
Method in class sbrt.kernel.math.stat.comp. MannWhitneyU 
 Computes and returns an approximate t value for the provided U value.
   getTau(double[], double[])   - 
Method in class sbrt.kernel.math.stat.comp. KendallTau 
 Computes and returns the correlation statistic for the provided values.
   getTau(Map<? extends V, Double>, Map<? extends V, Double>)   - 
Method in class sbrt.kernel.math.stat.comp. KendallTau 
 Computes and returns the correlation statistic for the provided values.
   getTau(Collection<Double>, Collection<Double>)   - 
Method in class sbrt.kernel.math.stat.comp. KendallTau 
 Computes and returns the correlation statistic for the provided values.
   getTau(double[], double[])   - 
Method in class sbrt.kernel.math.stat.comp. KendallTauA 
 Computes and returns the correlation statistic for the provided values.
   getTau(double[], double[])   - 
Method in class sbrt.kernel.math.stat.comp. KendallTauB 
 Computes and returns the correlation statistic for the provided values.
   getTermArray()   - 
Method in class sbrt.kernel.math. HashLinearComb 
 Returns the terms of this linear combination as an array.
   getTermArray()   - 
Method in interface sbrt.kernel.math. LinearComb 
 Returns the terms of this linear combination as an array.
   getTermArray()   - 
Method in interface sbrt.kernel.math. Polynomial 
 Returns an array containing all of the terms of this
 polynomial.
   getTermFormat()   - 
Method in class sbrt.shell.text.math. LinearCombFormatV1 
 Returns the format used for terms.
   getTermFormat()   - 
Method in class sbrt.shell.text.math. LinearCombFormatV2 
 Returns the format used for terms.
   getTermList()   - 
Method in class sbrt.kernel.math. AbstractLinearComb 
 Returns the terms of this linear combination as list.
   getTermList()   - 
Method in interface sbrt.kernel.math. LinearComb 
 Returns the terms of this linear combination as list.
   getTextFileFormat(String)   - 
Static method in class sbrt.shell.mng. AbstractProcessManager 
 Returns the text file format corresponding to the provided string.
   getTextFileFormat(ManagerInput<String, String>)   - 
Static method in class sbrt.shell.mng. AbstractProcessManager 
 Returns the parsed value for   AbstractProcessManager.FILE_FORMAT   in the provided input.
   getTieScore(Map<? extends V, Double>)   - 
Method in class sbrt.kernel.math.stat.comp. KendallTau 
 Computes and returns the tie score of the provided values.
   getTieScore(Collection<Double>)   - 
Method in class sbrt.kernel.math.stat.comp. KendallTau 
 Computes and returns the tie score of the provided values.
   getTieScore(double[])   - 
Method in class sbrt.kernel.math.stat.comp. KendallTau 
 Computes and returns the tie score of the provided values.
   getTolerance()   - 
Method in class sbrt.kernel.fba.comp. FbaOptComputer 
 Returns the allowable amount by which column variables
 may violate their defined constraints.
   getTolerance()   - 
Method in class sbrt.kernel.math. DoubleEquiv 
 Returns the tolerance used by this equivalence tester.
   getTolerance()   - 
Method in class sbrt.kernel.math. IntervalEquiv 
 Returns the tolerance used by this equivalence tester.
   getTolerance()   - 
Method in class sbrt.kernel.utilities.proc. IntervalComparison 
  
   getTolerance()   - 
Method in class sbrt.kernel.utilities.proc. VectorComparison 
  
   getTotal(IrrevRxnFluxome)   - 
Method in interface sbrt.kernel.fba.comp. ExtremeCurrentComputer 
 Returns the total number of operations required to compute the extreme
 currents of the provided fluxome.
   getTotal(IrrevRxnFluxome)   - 
Method in class sbrt.kernel.fba.comp. SlpExCurrComputerV2 
 Returns the total number of "operations" required to compute the extreme
 currents of the provided fluxome.
   getU()   - 
Method in class sbrt.kernel.math.stat.comp. MannWhitneyResults 
 Returns the computed U statistic.
   getU(List<? extends Number>, List<? extends Number>)   - 
Method in class sbrt.kernel.math.stat.comp. MannWhitneyU 
 Computes and returns the Mann-Whitney U statistic for the provided samples.
   getU(int[], int[])   - 
Method in class sbrt.kernel.math.stat.comp. MannWhitneyU 
 Computes and returns the Mann-Whitney U statistic for the provided samples.
   getU(double[], double[])   - 
Method in class sbrt.kernel.math.stat.comp. MannWhitneyU 
 Computes and returns the Mann-Whitney U statistic for the provided samples.
   getUnchangedVariables()   - 
Method in class sbrt.kernel.math.geom.comp. CdHarComputer.Results 
 Returns the variables whose values are the same as they were in the
 previous point.
   getUnions(Collection<Set<E>>)   - 
Method in class sbrt.kernel.math.comb.comp. SingleElementUnionsV1 
 Returns the set of single-element unions of the sets in the provided 
 collection.
   getUnions(Collection<Set<E>>)   - 
Method in class sbrt.kernel.math.comb.comp. SingleElementUnionsV2 
 Returns the set of single-element unions of the sets in the provided 
 collection.
   getUnions(Collection<Set<E>>)   - 
Method in class sbrt.kernel.math.comb.comp. StrictSingleElementUnions 
 Returns the set of strict single-element unions of the sets in the provided
 collection.
   getUpper()   - 
Method in exception sbrt.kernel.math. IllegalBoundsException 
 Returns the upper bound used to create this exception.
   getUpper()   - 
Method in class sbrt.kernel.math. Interval 
 Returns the upper bound (or endpoint) of this interval.
   getUpperBound(Node)   - 
Method in class sbrt.shell.text.fba. BiggRxnNodeParser 
 Returns the upper flux bound of the provided reaction node.
   getUpperBound(Node)   - 
Method in class sbrt.shell.text.fba. PalssonRxnNodeParser 
 Returns the upper flux bound of the provided reaction node.
   getValue()   - 
Method in exception sbrt.kernel.math. ConstraintViolationException 
 Returns the value that has violated its constraints.
   getValue1()   - 
Method in class sbrt.kernel.math. VectorComparer.Diff 
  
   getValue2()   - 
Method in class sbrt.kernel.math. VectorComparer.Diff 
  
   getValueFormat()   - 
Method in class sbrt.shell.text. ColonMapEntryFormatV1 
 Returns the format used for values.
   getValueFormat()   - 
Method in class sbrt.shell.text. EqualsMapEntryFormatV1 
 Returns the format used for values.
   getValueFormatter()   - 
Method in class sbrt.shell.text. AbstractMapFormat 
 Returns the formatter used for values.
   getValueParser()   - 
Method in class sbrt.shell.text. AbstractMapFormat 
 Returns the parser used for values.
   getVariable()   - 
Method in class sbrt.kernel.math. AbstractLinearComb.Term 
 Returns the variable contained in this term.
   getVariable()   - 
Method in exception sbrt.kernel.math.algebra. IllegalFreeVariableException 
 Returns the illegal free variable.
   getVariable()   - 
Method in exception sbrt.kernel.math. ConstraintViolationException 
 Returns the variable that has violated its constraints.
   getVariable()   - 
Method in exception sbrt.kernel.math. IllegalBoundsException 
 Returns the object with which these illegal bounds are associated.
   getVariable()   - 
Method in class sbrt.kernel.math. IntervalComparer.Diff 
  
   getVariable()   - 
Method in interface sbrt.kernel.math. LinearComb.Term 
 Returns the variable contained in this term.
   getVariable()   - 
Method in exception sbrt.kernel.math. MissingVariableException 
 Returns the missing variable.
   getVariable()   - 
Method in exception sbrt.kernel.math. UnboundedVariableException 
 Returns the unbounded variable.
   getVariable()   - 
Method in class sbrt.kernel.math. VectorComparer.Diff 
  
   getVariableFormat()   - 
Method in class sbrt.shell.text.math. LinCombTermFormatV1 
 Returns the format used for variables.
   getVariableFormat()   - 
Method in class sbrt.shell.text.math. LinCombTermFormatV2 
 Returns the format used for variables.
   getVariableFormat()   - 
Method in class sbrt.shell.text.math. LinearCombFormatV1 
 Returns the format used for variables.
   getVariableFormat()   - 
Method in class sbrt.shell.text.math. LinearCombFormatV2 
 Returns the format used for variables.
   getVariables()   - 
Method in class sbrt.kernel.math. AbstractLinearComb.Term 
 Returns the set of variables contained in this term.
   getVariables()   - 
Method in class sbrt.kernel.math.algebra. HashLinearSystemSolution 
 Returns the set of variables contained in this solution.
   getVariables()   - 
Method in interface sbrt.kernel.math.algebra. LinearSystemSolution 
 Returns the set of variables contained in this solution.
   getVariables()   - 
Method in interface sbrt.kernel.math.geom. ConvexPolytope 
 Returns the variables contained in the hyperplanes defining the bounding
 half-spaces.
   getVariables()   - 
Method in class sbrt.kernel.math.geom. HashConvexPolytope 
 Returns the variables contained in the hyperplanes defining the bounding
 half-spaces.
   getVariables()   - 
Method in class sbrt.kernel.math.geom. HashConvexPolytopeOld 
 Returns the variables contained in the hyperplanes defining the bounding
 half-spaces.
   getVariables()   - 
Method in class sbrt.kernel.math. HashLinearComb 
 Returns the set of variables contained in this
 mathematical expression.
   getVariables()   - 
Method in interface sbrt.kernel.math. MathExpr 
 Returns the set of variables contained in this
 mathematical expression.
   getVariables()   - 
Method in interface sbrt.kernel.math. Polynomial.Term 
 Returns the set of variables contained in this term.
   getVariance()   - 
Method in class sbrt.kernel.math.stat. NormalDist 
 Returns the variance of this distribution.
   getVarS(Map<? extends V, Double>, Map<? extends V, Double>)   - 
Method in class sbrt.kernel.math.stat.comp. KendallTau 
 Computes and returns the variance of S for the provided values.
   getVarS(Collection<Double>, Collection<Double>)   - 
Method in class sbrt.kernel.math.stat.comp. KendallTau 
 Computes and returns the variance of S for the provided values.
   getVarS(double[], double[])   - 
Method in class sbrt.kernel.math.stat.comp. KendallTau 
 Computes and returns the variance of S for the provided values.
   getVector(String)   - 
Static method in class sbrt.shell.mng.stat. CorrelationManager 
 Parses the file with the provided name and returns the vector it
 contains.
   getVector(String, PrintWriter)   - 
Static method in class sbrt.shell.mng.stat. CorrelationManager 
 Parses the file with the provided name, prints a status message to the
 provided print writer, and returns the vector the file contains.
   getVersion()   - 
Static method in class sbrt. Sbrt 
 Returns the full version number of the Systems Biology Research Tool.
   getXValues(ManagerInput<String, String>, PrintWriter)   - 
Static method in class sbrt.shell.mng.stat. CorrelationManager 
 Returns the parsed value for   CorrelationManager.X_FILE_NAME   in the provided input
 and prints a descriptive message to the provided print writer.
   getYValues(ManagerInput<String, String>, PrintWriter)   - 
Static method in class sbrt.shell.mng.stat. CorrelationManager 
 Returns the parsed value for   CorrelationManager.Y_FILE_NAME   in the provided input
 and prints a descriptive message to the provided print writer.
   getZeroCutoff()   - 
Method in class sbrt.kernel.fba.comp. AbstractFluxIntervalComputer 
 Returns the zero cutoff used by this flux interval computer.
   getZeroCutoff()   - 
Method in interface sbrt.kernel.fba.comp. FluxIntervalComputer 
 Returns the current zero cutoff.
   getZeroCutoff()   - 
Method in class sbrt.kernel.fba.comp. RxnDeletionComputer 
 Returns the current zero cutoff.
   getZeroCutoff()   - 
Method in class sbrt.kernel.fba.proc. CatalystDeletionProcess 
 Returns the current zero cutoff used by the reaction deletion computer.
   getZeroCutoff()   - 
Method in class sbrt.kernel.fba.proc. CvCatalystDeletionProcess 
 Returns the current zero cutoff used by the reaction deletion computer.
   getZeroCutoff()   - 
Method in class sbrt.kernel.fba.proc. CvRxnDeletionProcess 
 Returns the current zero cutoff used by the reaction deletion computer.
   getZeroCutoff()   - 
Method in class sbrt.kernel.fba.proc. RxnDeletionProcess 
 Returns the current zero cutoff used by the reaction deletion computer.
   getZeroCutoff()   - 
Method in class sbrt.kernel.math. AbstractLinearComb 
 Returns the current zero cutoff.
   getZeroCutoff()   - 
Method in interface sbrt.kernel.math. LinearComb 
 Returns the current zero cutoff.
   getZeroCutoff(ManagerInput<String, String>, PrintWriter)   - 
Static method in class sbrt.shell.mng. AbstractProcessManager 
 Returns the parsed value for   AbstractProcessManager.ZERO_CUTOFF   in the provided input.
   getZeroCutoff(ManagerInput<String, String>)   - 
Static method in class sbrt.shell.mng. AbstractProcessManager 
 Returns the parsed value for   AbstractProcessManager.ZERO_CUTOFF   in the provided input.
   GLPK   - 
Static variable in class sbrt.shell. ProgramSolverFactory 
 The string used to denote the GLPK program solver from GNU.
   GlpkSolver  < R , C > - Class in  sbrt.ext.glpk  This class is a wrapper for the GLPK package from GNU.   GlpkSolver()   - 
Constructor for class sbrt.ext.glpk. GlpkSolver 
 Constructs a new GLPK program solver.
   GREATER_THAN   - 
Static variable in class sbrt.shell.text. SpecialChars 
 >
   GUI_ARG   - 
Static variable in class sbrt. Sbrt 
 The argument used to launch the GUI from the command line.
   GZIP_SER   - 
Static variable in class sbrt.shell.io. FileFormat 
 Used to denote a file containing serialized objects
 that has been compressed using the GNU program gzip.
   GZIP_TEXT   - 
Static variable in class sbrt.shell.io. FileFormat 
 Used to denote a plain-text file that has been
 compressed using the GNU program gzip.
 
 


 
   
  
 
 
 
   
 
   
          Overview     
        Package   
        Class   
        Use   
          Tree     
          Deprecated     
       Index    
          Help     
   
 
 
  
 
 
 

 
  
   PREV LETTER   
   NEXT LETTER    
  
    FRAMES     
   NO FRAMES     
  
    All Classes  ');
  }
  //-->
 
 
    All Classes  
 


  
 
 
  
 

 A   B   C   D   E   F   G   H   I   J   K   L   M   N   O   P   Q   R   S   T   U   V   W   X   Y   Z
